# C9orf72 regulates the unfolded protein response and stress granule formation by interacting with eIF2α

**DOI:** 10.7150/thno.76138

**Published:** 2022-10-17

**Authors:** Wenzhong Zheng, Kexin Wang, Yachen Wu, Ge Yan, Chi Zhang, Zhiqiang Li, Lianrong Wang, Shi Chen

**Affiliations:** 1 Brain Center, Department of Neurosurgery, Ministry of Education Key Laboratory of Combinatorial Biosynthesis and Drug Discovery, Zhongnan Hospital of Wuhan University, School of Pharmaceutical Sciences, Wuhan University, Wuhan 430071, China; 2 Department of Gastroenterology, Hubei Clinical Center and Key Laboratory of Intestinal and Colorectal Disease, Taikang Center for Life and Medical Sciences, Zhongnan Hospital of Wuhan University, School of Pharmaceutical Sciences, Wuhan University, Wuhan 430071, China; 3 Department of Burn and Plastic Surgery, Shenzhen Institute of Translational Medicine, Health Science Center, the First Affiliated Hospital of Shenzhen University, Shenzhen Second People's Hospital, Shenzhen 518035, China

**Keywords:** C9orf72, eIF2α, translation initiation, unfolded protein response, stress granule formation

## Abstract

**Rationale:** A *C9orf72* hexanucleotide repeat expansion (GGGGCC) is the most common genetic origin of amyotrophic lateral sclerosis (ALS) and frontotemporal dementia (FTD). Haploinsufficiency of C9orf72 has been proposed as a possible disease mechanism (loss-of-function mechanism). Additionally, the aberrantly activated unfolded protein response (UPR) and stress granule (SG) formation are associated with the etiopathology of both ALS and FTD. However, the molecular determinants in this pathogenesis are not well characterized.

**Methods:** We performed an immunoprecipitation-mass spectrometry (IP-MS) assay to identify potential proteins interacting with the human C9orf72 protein. We used *C9orf72* knockout cell and rat models to determine the roles of C9orf72 in translation initiation and the stress response.

**Results:** Here, we show that C9orf72, which is genetically and pathologically related to ALS and FTD, interacts with eukaryotic initiation factor 2 subunit alpha (eIF2α) and regulates its function in translation initiation. *C9orf72* knockout weakens the interaction between eIF2α and eIF2B5, leading to global translation inhibition. Moreover, the loss of C9orf72 results in primary ER stress with activated UPR in rat spleens, which is one of the causes of splenomegaly with inflammation in *C9orf72*^-/-^ rats. Finally, C9orf72 delays SG formation by interacting with eIF2α in stressed cells.

**Conclusions:** In summary, these data reveal that C9orf72 modulates translation initiation, the UPR and SG formation, which have implications for understanding ALS/FTD pathogenesis.

## Introduction

Stress responses are important for homeostasis in eukaryotic cells when exposed to intrinsic or extrinsic stimuli, and the dysfunction of these responses may lead to many diseases, including neurodegenerative diseases. In mammals, the unfolded protein response (UPR) and stress granule (SG) formation are two critical types of stress responses. Under endoplasmic reticulum (ER) stress, the UPR can activate three different pathways, mediated by inositol-requiring kinase 1 (IRE1), activating transcription factor 6 (ATF6) and protein-kinase-like endoplasmic reticulum kinase (PERK) 1. Although the UPR, a conserved adaptive mechanism, assists in protein folding and degradation to maintain proper ER function, prolonged ER stress can promote apoptosis upon the sustained activation of certain irreversible signaling pathways 1. Accumulating evidence suggests that UPR activation is involved in the pathogenesis of amyotrophic lateral sclerosis (ALS) 2. SGs are cytoplasmic messenger ribonucleoprotein (mRNP) aggregates that assemble during cellular stress, requiring the phosphorylation of eukaryotic initiation factor 2 subunit alpha (eIF2α, encoded by the *EIF2S1* gene) 3. Under ER stress or upon exposure to other stress stimuli, PERK or other kinases (GCN2, PKR and HRI) phosphorylate eIF2α at serine 51, which rapidly inhibits global translation by suppressing the function of eukaryotic initiation factor 2B (eIF2B), the GDP/GTP exchange factor (GEF) of eukaryotic initiation factor 2 (eIF2) 4. In particular, disease-associated mutations that impair SG dynamics have been shown to play a causative role in ALS 5.

ALS is a motor neuron disease involving the progressive degeneration of upper and lower motor neurons in the nervous system 6. Frontotemporal dementia (FTD) is a common kind of presenile dementia associated with neuronal degeneration in patients' frontal and temporal lobes 7. Emerging evidence indicates that ALS and FTD overlap with each other, linked by pathology and genetics 8, 9. *C9orf72* hexanucleotide repeat expansion (GGGGCC) is the most typical genetic origin of ALS and FTD (C9ALS/FTD) 10-12 and has been suggested to cause neurodegeneration through three potential mechanisms: (1) the accumulation of sense (GGGGCC) and/or antisense (CCCCGG) repeat RNA 10, 13-15, (2) the accumulation of dipeptide repeat (DPR) proteins translated from repeat RNA 14-19, and (3) haploinsufficiency of the C9orf72 protein 10, 20-22. Evidence of the downregulation of *C9orf72* transcript expression in the neurons and nerve tissues of patients has been documented 10, 13, 20-22, suggesting that loss-of-function mechanisms may be important in C9ALS/FTD pathogenesis. In addition, *C9orf72* haploinsufficiency causes neurodegeneration in patient-derived motor neurons 23, and a reduced C9orf72 level induces cell hypersensitivity to stress 24. Resolving the functions of C9orf72 can help us understand the loss-of-function mechanisms of diseases such as ALS and FTD.

Alternative splicing of the human *C9orf72* gene generates two protein isoforms: a 222-amino acid (aa) short isoform encoded by *C9orf72* exons 2-5 and a 481-aa long isoform encoded by *C9orf72* exons 2-11 10, 25. Bioinformatics analyses of the C9orf72 long isoform have shown that this protein is a GEF for Rab GTPases containing a differentially expressed in normal and neoplasia (DENN) domain 26, 27. Due to the lack of most of the DENN domain and the complete dDENN (downstream DENN) domain, the C9orf72 short isoform, which has been shown to associate with components of the nuclear pore complex, seems to have different functions from the long isoform 25. Currently, most related studies focus on exploring the function of the C9orf72 long isoform. Some researchers have reported that the C9orf72 long isoform is an important regulator of endosomal and vesicle trafficking, as well as the autophagy-lysosome pathway 28-32. Moreover, an autophagy-associated complex of the C9orf72 long isoform and p62 clears SGs through arginine methylation 33. Additionally, *C9orf72* depletion results in autoimmune disease via the autophagy-lysosome pathway 34-36. The loss of *C9orf72* also induces inflammation in myeloid cells through STING activation 37. However, little is known about the association between the C9orf72 protein and the UPR or SG formation.

We conducted a preliminary study on *C9orf72* gene knockout (KO) human cell line and rat models to explore a new biological role for the human *C9orf72* gene and the rat *C9orf72* ortholog (*RGD1359108*, hereafter referred to as *C9orf72*). We found that C9orf72 interacts with eIF2α and its S51-phosphorylated form, maintaining the stability of the eIF2-eIF2B complex and regulating global translation. Importantly, C9orf72 acts as an effector of the eIF2α-related stress responses in the immune system and the nervous system. Thus, our work suggests that the newly identified C9orf72-eIF2 complex may be important for the pathogenesis of C9ALS/FTD.

## Materials and methods

### Plasmids

For GST-tagged protein expression, human *C9orf72* short, long and truncated (C9orf72^215-482^) isoform cDNAs were amplified by PCR and subcloned into the pGEX-6P-1 vector (GE Healthcare). For MBP-tagged protein expression, full-length human *EIF2S1* cDNA was cloned into the pMAL-c2x vector. To generate a vector expressing full-length human Ras-GTPase-activating protein (SH3 domain)-binding protein 1 (G3BP1), the *G3BP1* cDNA was cloned into the pCS2+ vector (CWBIO) with a C-terminal mCherry tag. The cDNAs encoding the human *C9orf72* short, long and truncated (C9orf72^215-482^) isoforms were cloned into the pcDNA 3.1+ vector containing an N-terminal Flag tag. The primers used to construct all these vectors are listed in Table S1.

### Rats and treatments

**Figure 1:**
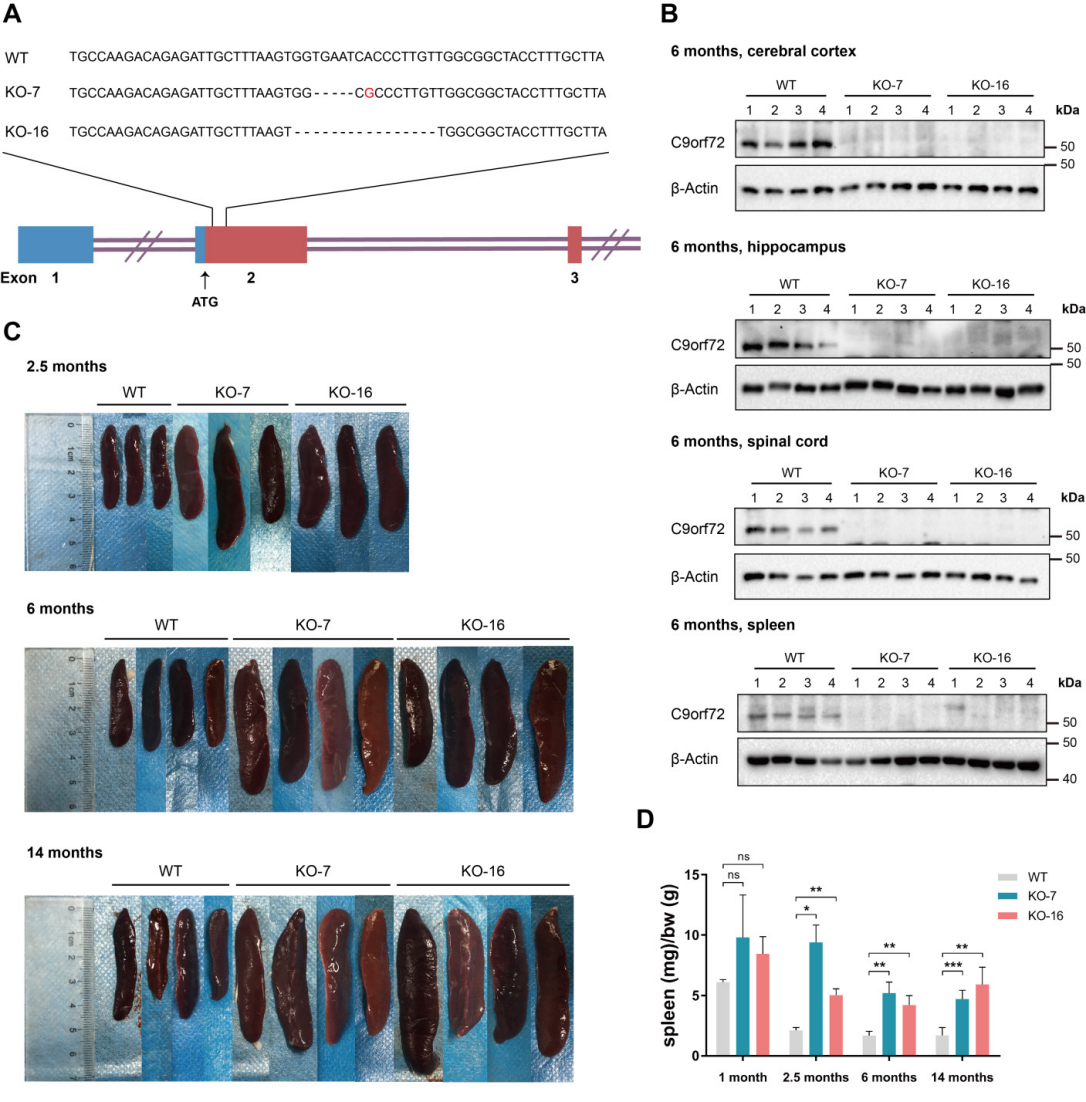
** C9orf72-null rats develop splenomegaly. (A)** Diagram of the rat *C9orf72* gene exons 1, 2 and 3 and the deleted sequences in two *C9orf72*-KO rat germlines (KO-7 and KO-16). The deleted sequences of *C9orf72*-null (KO-7 and KO-16) rats were located downstream of the ATG initiation codon in exon 2. **(B)** Western blot analyses of C9orf72 protein levels in the cerebral cortex, hippocampus, spinal cord and spleen of 6-month-old WT and *C9orf72*-null (KO-7 and KO-16) rats, with β-Actin serving as the loading control. A C9orf72 antibody (customized by GenScript) detected a band at 50 kDa in WT lysates that was not present in KO-7 and KO-16 lysates. **(C)** Gross images of splenomegaly (2.5 months, 6 months and 14 months of age, n = 3 or 4 rats of each genotype). **(D)** Spleen weights (in milligrams) normalized to body weight (in grams) at the indicated ages (n = 3 or 4 rats of each genotype, means ± SD, unpaired two-tailed t-test, n.s. *P* > 0.05, *P ≤ 0.05, **P ≤ 0.01, and ***P ≤ 0.001).

*C9orf72*-KO rats were generated on a Sprague-Dawley by Cyagen Biosciences Inc. (Guangzhou, China) using TALEN technology. TALEN target sites were designed in exon 2 of the rat *C9orf72* ortholog and the target TALEN sequences were: left, 5′-TTGCCAAGACAGAGATTGCTTT-3′, and right, 5′-TAAGCAAAGGTAGCCGCCAACA-3′. The KO rats were produced by microinjecting TALEN mRNAs into fertilized eggs from Sprague-Dawley strain. The sequences of the KO alleles have been validated. The following PCR primers were used for genotyping: forward, 5′-GGCTGAACCTAACCGCTGTTTTTGTA-3′, and reverse, 5′-CCACTCTCCGCATTCCGAAGAATT-3′. Two lines of KO rats were generated and named KO-7 and KO-16. KO-7 rats lack 5 bases (TGAAT) and include one point mutation (A to G), and KO-16 rats lack 16 bases (GGTGAATCACCCTTGT) (Figure 1A). Both KOs caused a frame shift and an early termination of C9orf72 protein translation. The mRNA transcribed from targeted allele with the frame shift undergoes nonsense-mediated decay. Male wild-type (WT) and *C9orf72*-KO rats were used in most experiments, unless indicated otherwise.

For 4-phenyl butyric acid (4-PBA) administration, 15-day-old male WT and *C9orf72*-KO rats were intraperitoneally injected with physiological saline or 4-PBA (250 mg/kg body weight, Sigma-Aldrich, P21005) for 30 consecutive days (once a day).

All animals were bred under humane care in the Center for Animal Experiment/Animal Biosafety Level-III Laboratory of Wuhan University, China. All experimental protocols were approved by the Institutional Animal Care and Use Committee at Wuhan University (IACUC NO. SQ20200283).

### Cell culture, transfection, and treatments

HEK-293T, HCT116 and HeLa cells were grown in DMEM (HyClone, SH30022.01) supplemented with 10% fetal bovine serum (FBS) (Zhejiang Tianhang Biotechnology, 11011-8611) and cultured in a 37°C incubator with 5% CO_2_.

For the SG induction experiments, HCT116 and HeLa cells were inoculated into 20 mm glass-bottom dishes. Up to 1 μg of the pCS2-G3BP1-mCherry plasmid was transfected into HCT116 cells, and the pCDNA3.1-Flag-tagged C9orf72 short isoform (C9S), C9orf72 truncated isoform (C9T) and C9orf72 long isoform (C9L) plasmids were transfected into HeLa cells after 24 h of culture using Lipofectamine 2000 reagent (Invitrogen, 11668019) according to the manufacturer's protocol. Approximately 24 h after transfection, two dishes from each group were induced with 0.2 mM sodium arsenite (SA; Xiya Reagent) in DMEM for 0.5 h. Then, one plate was immobilized with 4% paraformaldehyde for up to 10 min, and another was washed with PBS (HyClone, SH30256.01), recovered in complete DMEM for 1 h, and finally fixed with 4% paraformaldehyde.

Rat primary cerebral cortical neurons were isolated from the cerebral cortex of embryonic day 17.5 (E17.5) Sprague-Dawley rats (including WT, KO-7 and KO-16) as described previously 38, with some modification. Detached cortical neurons were inoculated into 24-well plates or 6-well plates coated with poly-D-lysine (Sigma-Aldrich, P0296) in Neurobasal medium (Gibco, 21103049) containing B27 supplement (Gibco, 17504044), L-glutamine (Gibco, 25030081), and penicillin-streptomycin (Gibco, 15070063). Cells were inoculated at a density of 8 × 10^4^ cells/well in 24-well plates for immunofluorescence staining or at 5 × 10^5^ cells/well in 6-well plates for western blotting and were then cultivated in a 37°C incubator with 5% CO_2_. After 9-11 days of incubation, the obtained neurons were used for experiments. For SG induction experiments, two dishes from each group were incubated with 0.05 mM SA (Xiya reagent) for 0.5 h. Then, neurons in one plate were fixed with 4% paraformaldehyde for up to 10 min, and neurons in the other plate were washed with PBS (HyClone, SH30256.01), recovered in complete Neurobasal medium for 1 h, and finally fixed with 4% paraformaldehyde.

In all SG experiments, at least 86 cells were counted in each experiment to quantify SG-containing cells. The SG clearance rate was calculated as follows: SG clearance rate = (1 - percentage of cells with SGs in the 0.5 h SA+1 h recovery group/percentage of cells with SGs in the 0.5 h SA group) × 100%.

### Surface sensing of translation (SUnSET) assay

The SUnSET assay was performed to assess global translation levels 39. HCT116 cells were incubated with fresh media containing 3 μg/mL puromycin (Beyotime, ST551-10 mg) or were treated with fresh media containing 0.2 mM SA (Xiya Reagent) and 3 μg/mL puromycin (Beyotime, ST551-10 mg) for 0.5 h at 37°C with 5% CO_2_. After the incubation, the untreated cells (Ctrl group), the cells treated with 3 μg/mL puromycin (Puromycin group) and the cells cotreated with 0.2 mM SA and 3 μg/mL puromycin (SA + puromycin group) were washed with PBS and lysed for western blot analysis.

### SDS-PAGE and western blotting

Whole cells and tissues were lysed in cell lysis buffer for western blotting and IP (Beyotime, P0013) with a 1% protease inhibitor cocktail (MedChemExpress, HY-K0010) and a 1% phosphatase inhibitor cocktail (Yeasen, 20109ES05) on ice, followed by quantification using the bicinchoninic acid assay (Beyotime, P0012).

For western blot analyses, after electrophoresis on SDS-PAGE gels, proteins were electrotransferred to PVDF membranes (Millipore, IPVH00010 and ISEQ00010) at 0.3 A for 1.5 h. Then, the membranes were blocked with 5% skim milk in TBST (25 mM Tris-HCl, 136 mM NaCl, and 0.05% Tween 20, pH=7.4) for 1 h and incubated with the primary antibody in TBST overnight at 4°C with gentle agitation. After washing with TBST three times, the membranes were incubated with an HRP-conjugated secondary antibody for up to 1 h at room temperature. Finally, after washing three times with TBST, we acquired images by applying the ECL substrate (Bio-Rad, 1705061) on the Bio-Rad ChemiDoc™ XRS+ system.

**Figure 2:**
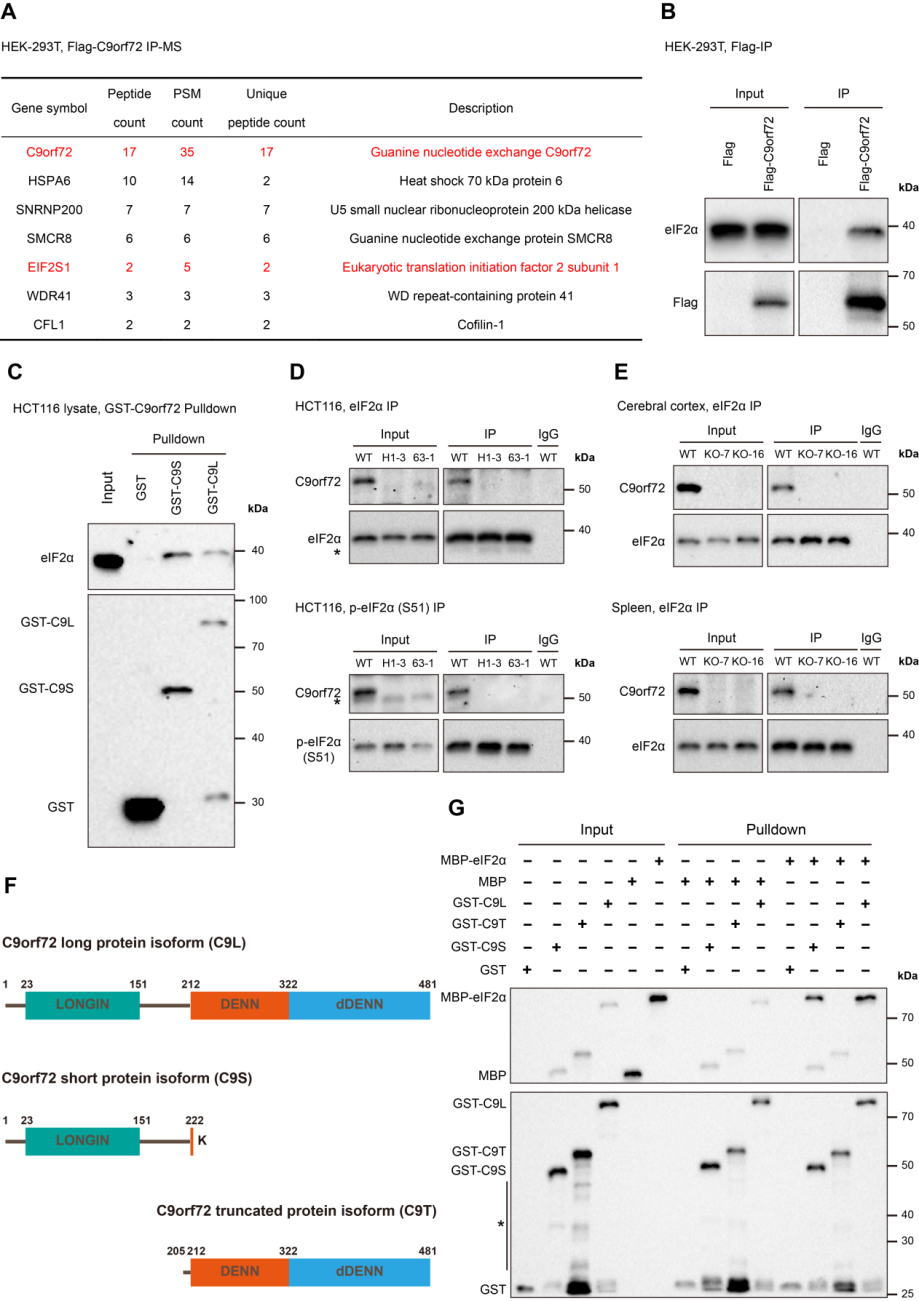
** C9orf72 interacts with eIF2α and p-eIF2α (S51) in the HCT116 cell line and rat tissues. (A)** *Flag-tag* or *Flag-C9orf72* (long version) was transfected into HEK-293T cells and IP was performed using a Flag antibody pre-coupled to Protein A/G magnetic beads, followed by an MS analysis. Potential Flag-C9orf72-associated proteins identified by MS are indicated in the table. The Flag-tag immunoprecipitated sample was used as a control. PSM, Peptide-Spectrum Match. **(B)** Lysates were prepared from HEK-293T cells overexpressing Flag-tag or Flag-C9orf72 (long isoform). Flag-tagged proteins were immunoprecipitated with a Flag antibody pre-coupled to Protein A/G magnetic beads followed by western blot analysis using antibodies against Flag or eIF2α. **(C)** GST-tag, GST-C9S or GST-C9L pre-bound to glutathione magnetic beads was incubated with WT HCT116 cell lysates followed by western blot analysis using antibodies against GST or eIF2α. **(D)** Endogenous eIF2α (top panel) or p-eIF2α (S51) (bottom panel) in WT or *C9orf72*^-/-^ (H1-3 and 63-1) HCT116 cell lines was immunoprecipitated with an eIF2α or p-eIF2α (S51) antibody pre-coupled to Protein A/G magnetic beads, followed by western blot analyses using antibodies against eIF2α, p-eIF2α (S51) or C9orf72 (from Proteintech). The asterisk (*) indicates a nonspecific band. **(E)** Endogenous eIF2α in the cerebral cortex (top panel) or spleen (bottom panel) tissue from WT or *C9orf72*-null (KO-7 and KO-16) rats was immunoprecipitated with an eIF2α antibody pre-coupled to Protein A/G magnetic beads followed by western blot analyses using antibodies against eIF2α or C9orf72 (from Proteintech). **(F)** Schematic of the C9orf72 protein isoforms used in this study, namely, C9orf72 long protein isoform (C9L), C9orf72 short protein isoform (C9S) and C9orf72 truncated protein isoform (C9T). **(G)** Western blot for GST-tagged recombinant C9orf72 protein isoforms and MBP-eIF2α from an *in vitro* pulldown assay. The asterisk (*) indicates a nonspecific band.

The primary and secondary antibodies employed for western blotting were as follows: anti-C9orf72 (customized by GenScript, used in Figure 1, Figure S1 and Figure S5), anti-C9orf72 (Proteintech, 25757-1-AP, used in Figure 2, Figure S7 and Figure S9), anti-GST (Immunoway, YM3144), anti-MBP-tag (Abclonal, AE016), anti-GRP78/BIP (Proteintech, 11587-1-AP), anti-ATF4 (Proteintech, 10835-1-AP), anti-eIF2α (Proteintech, 11170-1-AP), anti-eIF2B5 (Abclonal, A10263), anti-phospho-eIF2α (Ser51) (Cell Signaling Technology, 3398), anti-puromycin (Merck, clone 12D10, MABE343), anti-p62/SQSTM1 (Proteintech, 18420-1-AP), anti-DDDDK (Flag)-tag (Abclonal, AE005), anti-HA-tag (Abclonal, AE008), anti-BAX (Proteintech, 50599-2-Ig), anti-Caspase 3 (Proteintech, 19677-1-AP), anti-β-actin (Abclonal, AC026), goat anti-rabbit IgG (H + L)-HRP conjugate (Bio-Rad, 1706515), goat anti-mouse IgG (H + L)-HRP conjugate (Bio-Rad, 1706516), HRP-conjugated AffiniPure mouse anti-rabbit IgG light chain (Abclonal, AS061), and HRP-conjugated AffiniPure goat anti-mouse IgG light chain (Abclonal, AS062).

### RNA extraction, RT-PCR and RT-qPCR

After total RNA was extracted from cells and tissues with TRIzol according to the manufacturer's protocol (Life Technologies), cDNAs were synthesized using FastKing gDNA Dispelling RT SuperMix (TIANGEN, KR118) and at least three biological replicates from each group and at least two technical replicates were analyzed using AceQ qPCR SYBR Green Master Mix (Vazyme, Q111 and Q511) with a 7900HT fast Real-Time PCR system (Applied Biosystems). The ΔΔCt method was used to calculate mRNA fold changes, which were normalized to GAPDH expression. Differences between groups were evaluated with the unpaired two-tailed Student's t-test.

To detect the expression of *Cxcr1* by RT-PCR, cDNA was generated as described above. PCR assays were then set up using equal amounts of cDNA with 2× Rapid Taq Master Mix (Vazyme, P222) according to the manufacturer's protocols. The PCR cycling conditions were as follows: 95°C for 3 min; 32 cycles of 95°C for 15 s, 60°C for 15 s, and 72°C for 10 s; and 72°C for 5 min. The products were detected through 2% agarose gel electrophoresis.

All primer sequences employed for RT-PCR and RT-qPCR in this study are listed in Table S2.

### Immunofluorescence

For immunofluorescence assays, all cells were fixed in 4% paraformaldehyde for 10 min. Fixed cells were treated with cell permeabilization buffer (0.2% Triton X-100 in PBS) for 15 min and with cell blocking buffer (3% BSA in PBS) for up to 1 h at room temperature. All specimens were incubated with the primary antibodies at 4°C overnight. After washing three times with PBS, the specimens were incubated with a fluorochrome-conjugated secondary antibody at room temperature for 1.5 h. The specimens were washed three times with PBS, followed by an incubation with 0.5 μg/mL DAPI (Biofroxx, 1155MG010) in PBS for 10 min, and were ultimately imaged under a Nikon A1 confocal microscope after washes with PBS.

The following primary and secondary antibodies were used for immunofluorescence staining: anti-TUJ1 (Sigma-Aldrich, MAB1637), anti-G3BP1 (Proteintech, 13057-2-AP), anti-DDDDK (Flag)-tag (Abclonal, AE005), anti-ChAT (Santa Cruz, sc-55557), anti-TDP-43 (Proteintech, 10782-2-AP), DyLight 488-conjugated goat anti-rabbit IgG (Abbkine, A23220), and DyLight 549-conjugated goat anti-mouse IgG (Abbkine, A23310).

### Immunoprecipitation (IP)

HEK-293T or HeLa cells were transfected with plasmids, as indicated in the figures, and cultured for 24 hours before collecting the protein lysate. All cells were washed with precooled PBS before collection and lysed in cell lysis buffer for western blotting and IP (Beyotime, P0013) supplemented with a 1% protease inhibitor cocktail (MedChemExpress, HY-K0010) and a 1% phosphatase inhibitor cocktail (Yeasen, 20109ES05) for up to 30 min at 4°C, after which they were centrifuged at 12,000 g for 10 min. Rat cerebral cortex and spleen samples were homogenized in cell lysis buffer with an electric homogenizer, lysed for 1 h at 4°C, and centrifuged at 12,000 g for 10 min at 4°C. The collected supernatants were used for IP using a previously described protocol 40.

The antibodies used for IP were as follows: anti-eIF2α (Proteintech, 11170-1-AP), anti-phospho-eIF2α (Ser51) (Cell Signaling Technology, 3398), and anti-DDDDK (Flag)-tag (Abclonal, AE092).

### Recombinant protein expression and purification

GST-tag, the GST-tagged C9orf72 short protein isoform (GST-C9S), the C9orf72 truncated protein isoform (GST-C9T), and the C9orf72 long protein isoform (GST-C9L) as well as MBP-tag and MBP-tagged eIF2α (MBP-eIF2α) were expressed in Rosetta *E. coli* cells. Precultures were grown overnight in 20 mL of LB containing ampicillin at 37°C. Then, 1 L expanded cultures were grown until OD_600_=0.6 at 37°C. Following induction with 0.1 mM IPTG (Yeasen, 10902ES08), the cultures were further incubated at 16°C overnight. Centrifugally collected cells were resuspended in binding buffer (20 mM Tris, 200 mM NaCl, 1 mM EDTA, 1 mM DTT and 1% protease inhibitor cocktail, pH=7.4), crushed with a high-pressure cell crusher (Union-biotech, UH-03), and finally centrifuged for 30 min at 20,000 g. MBP-tag and MBP-eIF2α were purified using dextrin beads 6FF (Smart-lifesciences, SA026010), and all GST-tagged proteins were purified with glutathione magnetic beads (Smart-lifesciences, SM00201) and frozen at -80°C in buffer containing 20 mM Tris-HCl, pH=7.4, 150 mM NaCl and 20% glycerol.

### GST pulldown assay

Total cell lysates were prepared as described above. Thirty microliters of glutathione magnetic beads (Smart-lifesciences, SM00201) were mixed with 1 µg of recombinant GST, GST-C9S or GST-C9L in 400 µL of PBS (HyClone, SH30256.01) and then incubated for up to 2 h at 4°C. After four washes with cold wash buffer A (20 mM Tris-HCl, pH 7.5, 250 mM NaCl, 0.3% Triton X-100 and 1% protease inhibitor cocktail), the protein-bound beads were incubated with 1 mL of cell lysate by end-over-end mixing for up to 4 h at 4°C. The mixtures were washed four times with precooled wash buffer B (20 mM Tris-HCl, pH 7.5, 400 mM NaCl, 0.6% Triton X-100 and 1% protease inhibitor cocktail).

Purified GST or GST-tagged proteins (0.5 µg) were bound to 15 µL of glutathione magnetic beads in PBS for up to 2 h at 4°C. The protein-bound beads were washed four times with cold wash buffer A (20 mM Tris-HCl, pH 7.5, 250 mM NaCl, 0.3% Triton X-100 and 1% protease inhibitor cocktail) and incubated with 0.5 µg of purified MBP or MBP-eIF2α for 4 h at 4°C. The mixtures were washed six times with wash buffer B (20 mM Tris-HCl, pH 7.5, 400 mM NaCl, 0.5% Triton X-100 and 1% protease inhibitor cocktail).

Finally, the bound proteins were analyzed by western blotting.

### Statistical analysis

All data are presented as mean values ± standard deviation (SD), and n indicates the number of biological replicates in each experiment. Using GraphPad Prism 7 software, statistical analyses were performed with unpaired two-tailed t-test, one-way ANOVA or two-way ANOVA. Detailed information about each statistical analysis is provided in the relevant figure legends.

## Results

### *C9orf72*-null rats exhibit splenomegaly

We generated two rat lines with TALEN-mediated *C9orf72* KO, designated KO-7 (missing 5 bases, with one point mutation) and KO-16 (missing 16 bases), to better study the function of the C9orf72 protein (Figure 1A). We confirmed *C9orf72* KO by generating an anti-C9orf72 antibody (customized by GenScript) against a 14-aa polypeptide (corresponding to aa 194-207 of human C9orf72). The knockout of the *C9orf72* gene in KO-7 and KO-16 rats caused the loss of C9orf72 protein (Figure 1B; Figure S1A). Heterozygous intercross breeding of *C9orf72* KO rats yielded the expected Mendelian ratio, and *C9orf72* KO rats had a normal life span (Figure S1B, C).

*C9orf72*-null rats from both lines developed visibly enlarged spleens at 2.5, 6 and 14 months of age, and the sizes of the spleens of *C9orf72*-null rats from both lines slowly increased with age (Figure 1C). The normalized spleen weights of *C9orf72*-null rats increased significantly in the three age groups (Figure 1D). In addition, *C9orf72*-null rats developed enlarged cervical lymph nodes (Figure S1D, E).

We then performed ethology examinations to determine whether *C9orf72* KO led to abnormal characteristics. Front paw strength, rotarod performance, locomotor coordination and endurance exercise performance were similar in WT and *C9orf72*-null rats (Figure S2A-D). These results suggested that the loss of *C9orf72* in rats did not influence locomotion. In addition, the elevated plus-maze test revealed no signs of anxiety in *C9orf72*-null rats compared with WT rats (Figure S2E). Open-field observations did not indicate a difference in locomotor behaviors or zone preference between WT and *C9orf72*-null rats at the ages of 3 months and 12 months (Figure S2F), revealing no significant change in locomotion or emotion in *C9orf72*-null rats.

### C9orf72 interacts with eIF2α in the human cell line and rat tissues

We performed an immunoprecipitation-mass spectrometry (IP-MS) assay to identify potential proteins interacting with the human C9orf72 long protein isoform. IP studies were performed with Flag antibody using the lysates of HEK-293T cells transfected with *Flag-tag* or *Flag-C9orf72* (long version), followed by the MS analysis of the immunoprecipitated samples. Here, the Flag-tag immunoprecipitated sample was used as a control. This analysis identified three known C9orf72-binding partners, SMCR8, WDR41 and CFL1, and a new C9orf72-binding protein, eIF2α (Figure 2A). We also confirmed that C9orf72 interacted with eIF2α by performing cell transfection and co-immunoprecipitation (co-IP) experiments (Figure 2B). Moreover, the GST pulldown assay revealed that eIF2α interacted with both the C9orf72 short protein isoform (C9S) and the C9orf72 long protein isoform (C9L) (Figure 2C).

Next, we generated two *C9orf72*-KO HCT116 cell lines (named H1-3 and 63-1) using CRISPR/Cas9 technology (Figure S3). Endogenous eIF2α and p-eIF2α (S51) co-IP experiments indicated that C9orf72 interacted with eIF2α and p-eIF2α (S51) in WT HCT116 cells (Figure 2D). The immunoprecipitates from two *C9orf72*-KO HCT116 cell lines were used as controls (Figure 2D). At the same time, the interaction between C9orf72 and eIF2α was also detected in WT rat cerebral cortex and spleen tissues (*C9orf72*-KO rat immunoprecipitated samples were used as controls) (Figure 2E). We co-transfected HEK-293T cells with Flag-C9orf72 and HA tagged wild-type eIF2α, nonphosphorylatable eIF2α (S51A) or phosphomimetic eIF2α (S51D) and then performed co-IP assays to investigate the binding preference of C9orf72 for the nonphosphorylated or phosphorylated eIF2α form. The interaction of C9orf72 with the eIF2α (S51A) variant was obviously stronger than that of C9orf72 with the eIF2α (S51D) variant (Figure S4), suggesting that phosphorylation of eIF2α at serine 51 weakened the binding of C9orf72 to eIF2α.

Finally, we further purified a truncated C9orf72 protein isoform (C9T) containing the complete DENN-dDENN domain (Figure 2F) and MBP-tagged eIF2α to confirm the direct interaction of the two C9orf72 protein isoforms with eIF2α and to test which domains were necessary for the interaction. Strikingly, only recombinant GST-C9S and GST-C9L interacted with MBP-eIF2α (Figure 2G). Taken together, these results indicated that C9orf72 interacts with eIF2α and p-eIF2α (S51) and that the C9orf72 short protein isoform containing a LONGIN domain is necessary for the interaction with eIF2α.

### Loss of C9orf72 weakens the interaction of eIF2α with eIF2B5, leading to global translation repression

The eIF2B complex binds eIF2 and initiates translation by converting eIF2 from an inert GDP-bound state to an active GTP-bound state 4. Phosphorylation at Ser 51 of the eIF2α subunit results in a stable interaction with eIF2B and interferes with eIF2-GDP/GTP exchange, mediating a decline in overall translation initiation 4. Hence, regulating the interaction of eIF2 with eIF2B has a direct effect on eIF2-related translation initiation.

**Figure 3:**
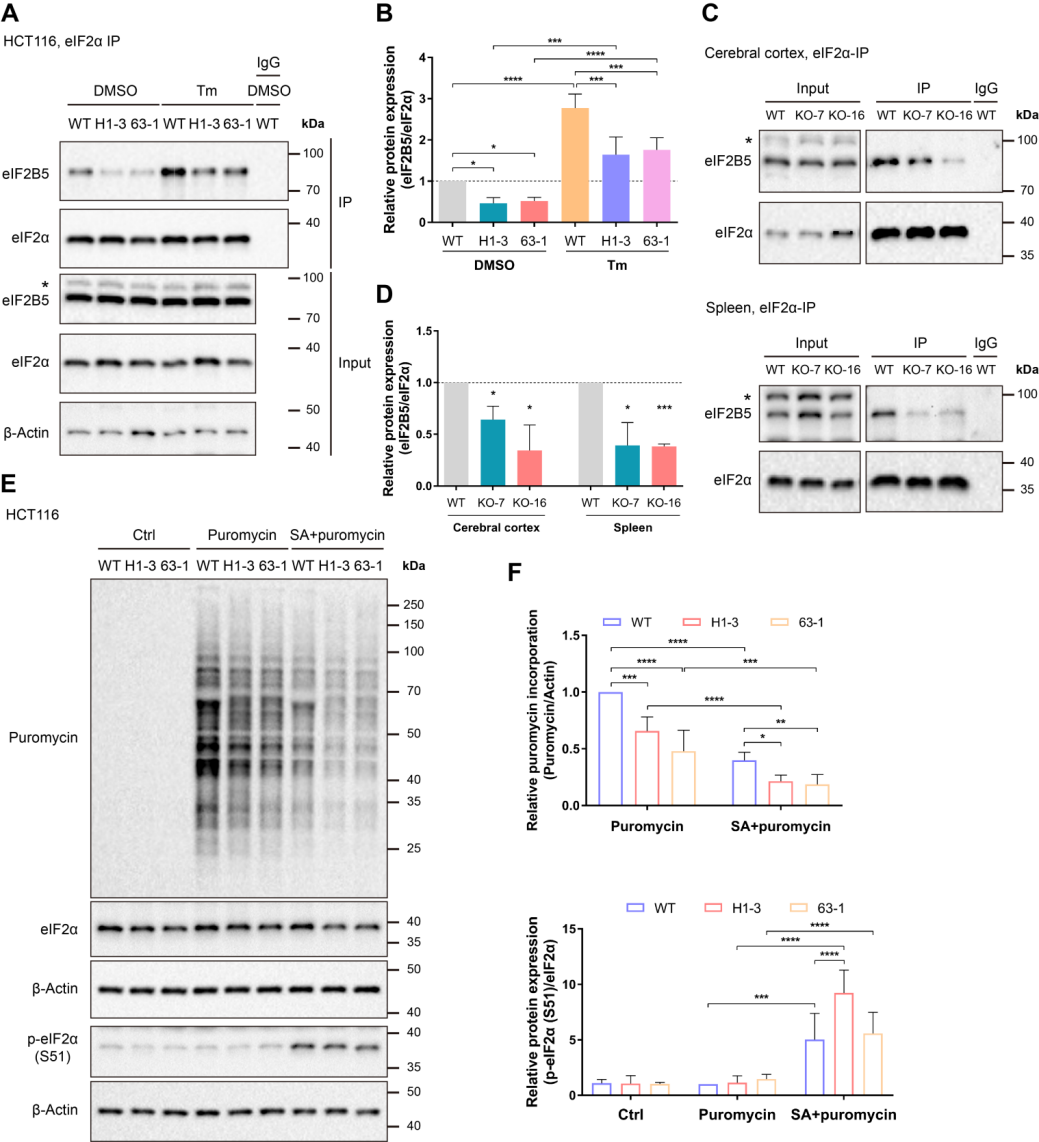
** Loss of C9orf72 weakens the interaction of eIF2α with eIF2B5 and inhibits global translation. (A)** Lysates were prepared from either the DMSO- or Tm (1 μg/mL for 24 h)-treated WT or *C9orf72*^-/-^ (H1-3 and 63-1) HCT116 cell lines. Endogenous eIF2α was immunoprecipitated with an eIF2α antibody pre-coupled to Protein A/G magnetic beads followed by western blot analyses using antibodies against eIF2α or eIF2B5. The asterisk (*) indicates a nonspecific band. **(B)** Relative ratio of eIF2B5 to eIF2α based on the western blot results (A) (n = 3 independent experiments, means ± SD, two-way ANOVA with Fisher's LSD test, *P ≤ 0.05, ***P ≤ 0.001, and ****P ≤ 0.0001). **(C)** Endogenous eIF2α in the cerebral cortex (top panel) or spleen (bottom panel) tissue from WT or *C9orf72*-null (KO-7 and KO-16) rats was immunoprecipitated with an eIF2α antibody pre-coupled to Protein A/G magnetic beads followed by western blot analyses using antibodies against eIF2α or eIF2B5. The asterisk (*) indicates a nonspecific band. **(D)** Relative ratio of eIF2B5 to eIF2α based on the western blot results (C) (n = 3 independent experiments, means ± SD, unpaired two-tailed t-test, *P ≤ 0.05 and ***P ≤ 0.001). **(E)** Western blot analysis of puromycin incorporation and eIF2α and p-eIF2α (S51) protein levels in the Ctrl group (untreated HCT116 cell lines, negative control), Puromycin group (HCT116 cell lines treated with 3 μg/mL puromycin for 30 min) and SA + puromycin group (HCT116 cell lines cotreated with 0.2 mM SA and 3 μg/mL puromycin for 30 min). β-Actin was used as a loading control. **(F)** Quantification of puromycin incorporation (upper panel) and the relative ratio of p-eIF2α (S51) to eIF2α (lower panel) based on the western blot results (E) (n = 4 independent experiments, means ± SD, two-way ANOVA with Fisher's LSD test, *P ≤ 0.05, **P ≤ 0.01, ***P ≤ 0.001, and ****P ≤ 0.0001).

We performed co-IP studies to detect the interaction of eIF2α with eIF2B5 in both normal HCT116 cells and cells under ER stress and to examine whether the loss of C9orf72 can affect the functions of eIF2α. eIF2B5, an essential component of the eIF2B complex, contains a GEF domain and a region that interacts with eIF2α 41. First, tunicamycin (Tm) induced ER stress in HCT116 cell lines, as measured by the increased mRNA expression of *GRP78*, *ATF4* and *CHOP,* as well as the expression of the GRP78 and ATF4 proteins (Figure S5A-C). Moreover, the co-IP analyses indicated that the loss of C9orf72 weakened the interaction of eIF2α with eIF2B5 in both normal HCT116 cells and cells under ER stress (Figure 3A, B). Likewise, the interaction between eIF2α and eIF2B5 was weakened in the cerebral cortex and spleen of *C9orf72*-null rats (Figure 3C, D). However, the co-IP assay revealed that eIF2B5 was not pulled down together with eIF2α when immunoprecipitating Flag-C9orf72 (Figure S6), suggesting that C9orf72 controlled the interaction of eIF2α with eIF2B5 by interacting with eIF2α instead of eIF2B5. Furthermore, we reconstituted *C9orf72* KO HCT116 cells with Flag-tagged C9orf72 isoforms, as indicated in the schematic diagram (Figure 2F), and examined the interaction of eIF2α with eIF2B5. Only reconstitution with C9S or C9L, which interacted with eIF2α, restored the interaction of eIF2α with eIF2B5 in *C9orf72* KO cells (Figure S7A, B). Overall, these results suggest a repression of eIF2B GEF activity toward eIF2 in *C9orf72*-null cell lines and rat tissues, which may lead to ER stress or SG-like translation inhibition.

Given that the loss of C9orf72 weakened the interaction of eIF2α with eIF2B5, we hypothesized that the loss of C9orf72 could impair the function of the eIF2-eIF2B complex in translation initiation and repress global protein synthesis. To test this hypothesis, the SUnSET assay was used to quantify global translation levels in unstressed and arsenite-stressed HCT116 cell lines 39. Here, cells treated with sodium arsenite (SA) were used as positive controls because SA can induce oxidative stress and HRI activation, leading to eIF2α phosphorylation and global translation repression 3, 42. The SA-induced increase in eIF2α phosphorylation was accompanied by a comparable reduction in overall protein translation compared with the levels in unstressed HCT116 cells (Figure 3E, F). Importantly, the loss of C9orf72 inhibited global translation in both unstressed and stressed HCT116 cells, as measured by reduced puromycin incorporation (Figure 3E, F). We tested the roles of C9orf72 isoforms in translation initiation by reconstituting *C9orf72* KO HCT116 cells with Flag-tagged C9orf72 isoforms and examining puromycin incorporation. Only reconstitution with C9S or C9L, which interacted with eIF2α, restored the global translation levels of *C9orf72* KO cells (Figure S7C, D). The weak interaction of eIF2α with eIF2B5 and the reduced global translation efficiency might sensitize *C9orf72^-/-^* cells to some stresses, such as arsenite stress.

### Loss of C9orf72 induces primary ER stress in the cerebral cortex, hippocampus and spleen

**Figure 4:**
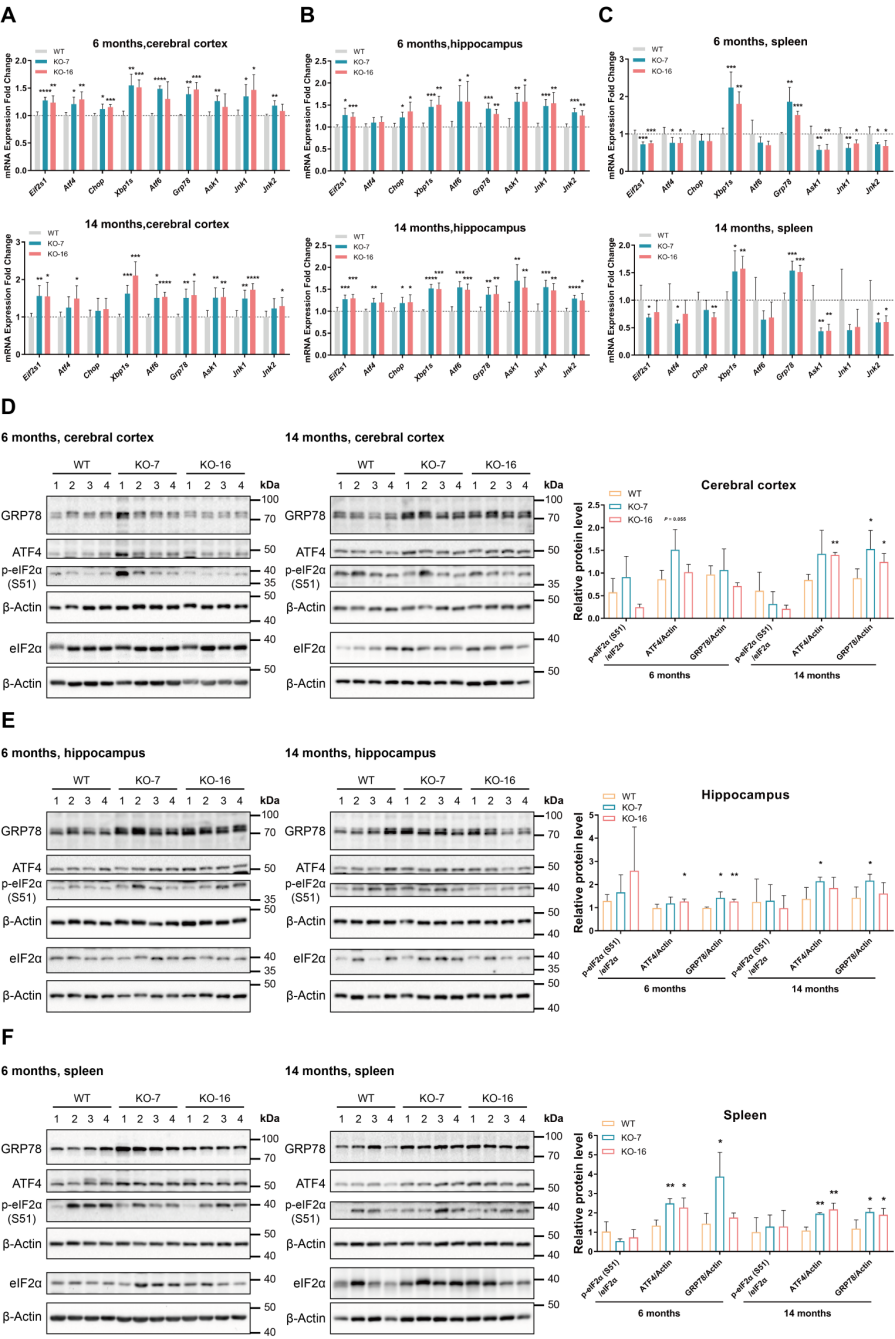
**Loss of C9orf72 induces ER stress in the cerebral cortex, hippocampus and spleen. (A-C)** Analyses of the mRNA expression of UPR-related genes in the cerebral cortex (A), hippocampus (B), and spleen (C) from male WT, KO-7 and KO-16 rats at 6 months (top panels) and 14 months (bottom panels) (n = 5 or 6 rats of each genotype, means ± SD, unpaired two-tailed t-test, *P ≤ 0.05, **P ≤ 0.01, ***P ≤ 0.001, and ****P ≤ 0.0001). **(D-F)** Western blot analyses of ER stress marker (ratio of p-eIF2α (S51) to eIF2α, ATF4 and GRP78) levels in cerebral cortex (D), hippocampus (E), and spleen (F) samples from male WT, KO-7 and KO-16 rats at 6 months (left panels) and 14 months (middle panels). The quantification of the western blot results is shown in the charts on the right (n = 4 rats of each genotype, mean ± SD, unpaired two-tailed t-test, *P ≤ 0.05 and **P ≤ 0.01). β-Actin was used as a loading control.

Since eIF2α is a key factor in the PERK-eIF2 branch of the UPR and an interaction between C9orf72 and eIF2α has been detected (Figure 2), we specifically measured the transcript levels of UPR-related genes using RT-qPCR. Here, we observed an emerging state of ER stress in the cerebral cortex of 6-month-old *C9orf72*-KO rats that was characterized by the upregulation of *Eif2s1*, *Atf4*, *Xbp1s*, *Grp78* and *Jnk1* (Figure 4A). Furthermore, *Eif2s1*, *Xbp1s*, *Atf6*, *Grp78*, *Ask1* and *Jnk1* mRNA expression increased in the cerebral cortex of 14-month-old *C9orf72*-KO rats (Figure 4A). By 6 months and 14 months of age, levels of the *Eif2s1*, *Chop*, *Xbp1s*,* Atf6*, *Grp78*, *Ask1*, *Jnk1* and *Jnk2* transcripts were increased to varying degrees in the hippocampus, indicating primary ER stress (Figure 4B). In the spleen, we observed the significant upregulation of *Xbp1s* and *Grp78,* as well as the downregulation of *Ask1* and *Jnk1/2*, suggesting the activation of the GRP78-IRE1-XBP1 branch rather than the GRP78-IRE1-ASK1-JNK branch (Figure 4C).

Additionally, at the ages of 6 months and 14 months, the western blot analyses of ER stress markers showed the upregulation of ATF4 and GRP78 in three *C9orf72*-KO rat tissues (Figure 4D-F). However, at 6 months and 14 months of age, we did not observe obvious differences in UPR-related mRNA and protein expression in the spinal cord (Figure S8). Altogether, these data indicate that the loss of C9orf72 in rats leads to primary ER stress in the cerebral cortex, hippocampus and spleen but not in the spinal cord.

### ER stress induced by Tm increases apoptosis in *C9orf72*-null rat primary cerebral cortical neurons

As shown in Figure S9A and B, *C9orf72* KO increased the number of apoptotic primary neurons compared to WT primary neurons when treated with Tm. The increased Tm-induced apoptosis of the primary cerebral cortical neurons from *C9orf72*-null rats was relieved by the ER stress inhibitor 4-PBA (Figure S9A, B), suggesting that the primary neuron apoptosis induced by Tm was caused by ER stress. Similar results were obtained from the neutral red uptake (NRU) assay in HCT116 cells incubated with Tm and/or 4-PBA (Figure S9C).

As expected, Tm-induced ER stress resulted in the upregulation of the ER stress markers GRP78 and ATF4 in the WT, KO-7 and KO-16 Tm-treated groups, which was suppressed by 4-PBA (Figure S9D, E). Moreover, the higher levels of cleaved Caspase3 and BAX were detected in the Tm-induced KO-7 and KO-16 groups, and these changes were relieved by 4-PBA (Figure S9D, E). Based on these results, *C9orf72* KO neurons were more sensitive to ER stress, consistent with the data presented in Figure S9A and B. In brief, C9orf72 could protect cells from prolonged ER stress.

### Inhibition of ER stress by 4-PBA treatment reverses the immunophenotype in the *C9orf72*-null spleen

**Figure 5:**
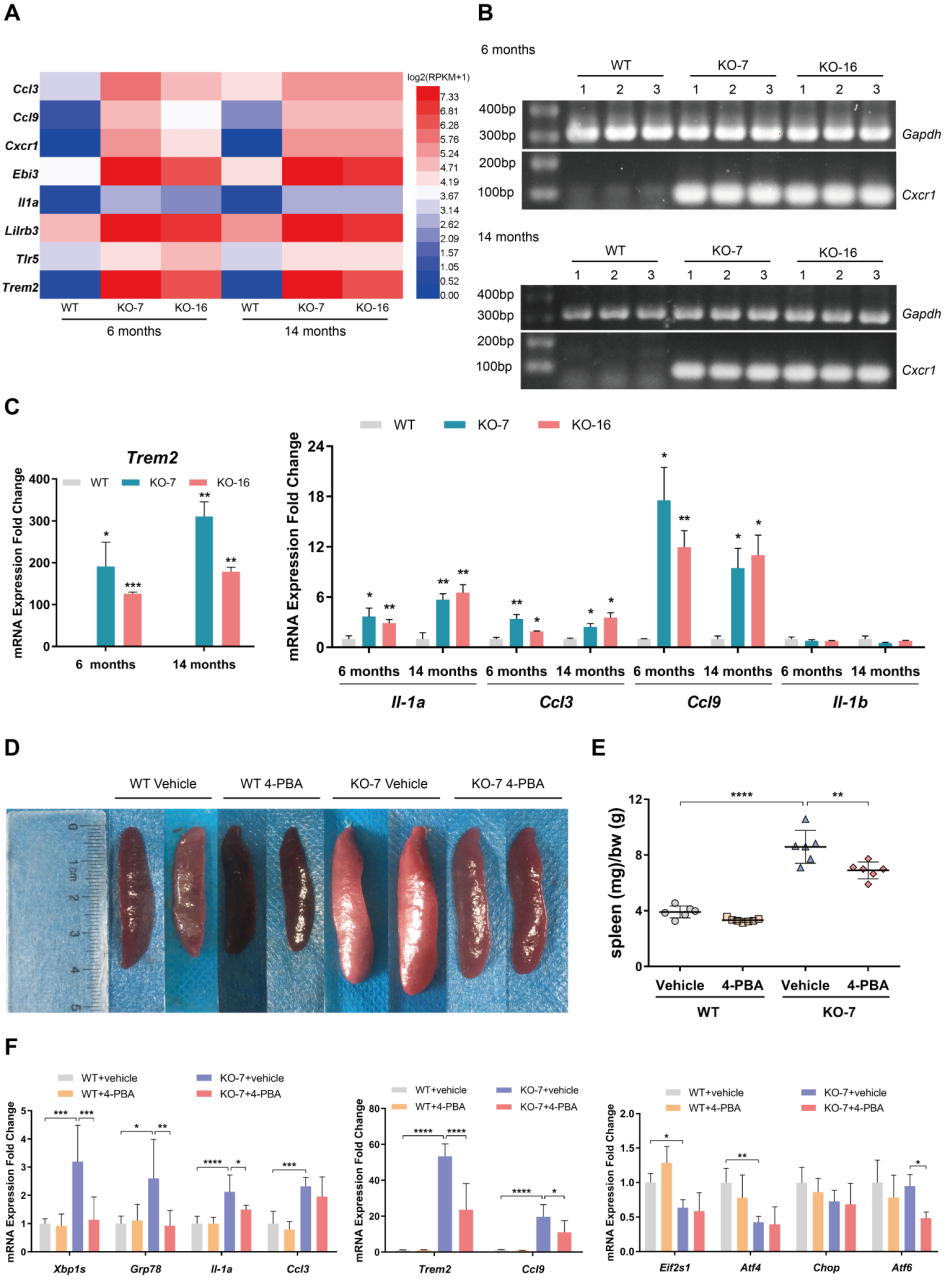
** Inhibition of ER stress by 4-PBA treatment reverses the immunophenotype of *C9orf72*-null spleen. (A)** Heatmap showing the expression (log2(x + 1)-transformed RPKM values) of some immune receptor and inflammatory cytokine genes in 6-month-old and 14-month-old rat spleens. **(B)** RT-PCR analysis of the *Cxcr1* mRNA level. **(C)** The mRNA expression levels of *Trem2*, *Ccl3*, *Ccl9*, *Il-1a* and *Il-1b* were detected using RT-qPCR (n = 3 rats of each genotype, means ± SD, unpaired two-tailed t-test, *P ≤ 0.05, **P ≤ 0.01, and ***P ≤ 0.001). **(D, E)** Typical images of the spleens (D) and spleen weights (in milligrams) normalized to body weight (in grams) (E) in 45-day-old male WT and *C9orf72*-KO rats after 30 days of the intraperitoneal injection of vehicle or 4-PBA (n = 6 or 7 rats in each group, means ± SD, two-way ANOVA and followed by Tukey's post hoc test, **P ≤ 0.01 and ****P ≤ 0.0001). **(F)** Analyses of the mRNA expression of *Xbp1s*, *Grp78* and other ER stress markers, as well as the immune-related genes *Trem2*, *Ccl3*, *Ccl9* and *Il-1a*, in the spleens of rats treated with either vehicle or 4-PBA (n = 6 or 7 rats in each group, means ± SD, two-way ANOVA and followed by Tukey's post hoc test, *P ≤ 0.05, **P ≤ 0.01, ***P ≤ 0.001, and ****P ≤ 0.0001).

Inflammation is considered an essential process in the development of ALS and FTD. As the *C9orf72*-null rats exhibited splenomegaly and cervical lymphadenopathy (Figure 1C, D; Figure S1D, E), which is commonly associated with diseases such as chronic lymphocytic leukemia 43, we investigated the expression of immune receptor and inflammatory cytokine genes using RNA-seq, RT-PCR and RT-qPCR assays. The RNA-seq heatmap of data from 6-month-old and 14-month-old rat spleens showed significant upregulation of the expression of some immune receptor (*Cxcr1*, *Trem2* and *Tlr5*) and inflammatory cytokine (*Ccl3*, *Ccl9*, *Ebi3*, *Il-1a* and *Lilrb3*) genes in *C9orf72*-null rats (Figure 5A). A substantial increase in the mRNA expression of the macrophage and monocyte receptor gene *Trem2* was observed in *C9orf72*^-/-^ spleens, along with the chemokine receptor gene *Cxcr1* (Figure 5B, C). Significant increases in the expression levels of the inflammatory cytokine genes *Ccl3*,* Ccl9* and *Il-1a* were also detected using RT-qPCR (Figure 5C). Here, *Il-1b* served as the negative control, which presented no change in mRNA expression according to the RNA-seq and RT-qPCR results (Figure 5C). These results suggested that *C9orf72*-null rats developed progressive splenomegaly with inflammation.

Accumulating evidence now shows that ER stress contributes to disorders of the immune system 44. Notably, the IRE1-XBP1 branch of the UPR plays an important role in immune responses by regulating the development, differentiation, function and survival of immune cells 44. The compound 4-PBA relieves ER stress in different tissues, including the spleen 45. We administered an intraperitoneal injection of 4-PBA into 15-day-old male WT and *C9orf72*-null rats for 30 days to examine whether the immunophenotype observed in *C9orf72*-null rats resulted from ER stress. As expected, at the age of 45 days, spleens of the control *C9orf72*-null rats showed increases in both size and normalized weight compared to those of the control WT rats (Figure 5D, E). 4-PBA administration did not significantly change the spleen size or normalized weight in WT rats (Figure 5D, E). In contrast, the same treatment obviously decreased the normalized spleen weight and size in *C9orf72*-null rats (Figure 5D, E).

Furthermore, a significant downregulation of *Xbp1s* and *Grp78* mRNA levels was observed in the spleens of KO rats treated with 4-PBA, but no changes in *Eif2s1*, *Atf4* and *Chop* mRNA expression were detected after 4-PBA treatment, showing that 4-PBA alleviated ER stress by reducing the activation of the GRP78-IRE1-XBP1 pathway in *C9orf72*-null spleens (Figure 5F). After relieving ER stress in *C9orf72*-null spleens, the RT-qPCR analysis of spleens from 4-PBA-treated rats showed obvious decreases in the expression of the macrophage marker gene *Trem2* and inflammatory cytokine genes, including *Il-1a* and *Ccl9* (Figure 5F). Collectively, these results indicated that 4-PBA treatment reversed the immunophenotype found in the spleens of *C9orf72*-null rats, suggesting that the immunophenotype is a consequence of primary ER stress in *C9orf72*-null spleens.

### C9orf72 regulates stress granule formation by interacting with eIF2α

**Figure 6:**
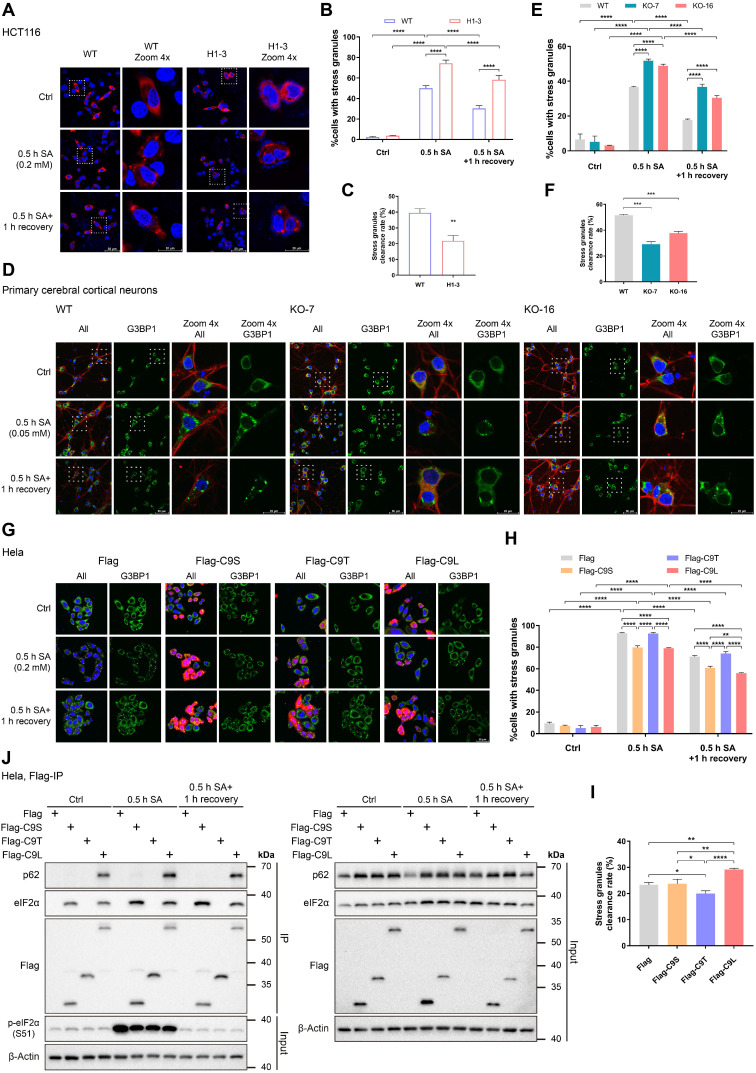
** C9orf72 regulates stress granule formation by interacting with eIF2α. (A)** Images of immunofluorescence staining of WT and *C9orf72^-/-^* (H1-3) HCT116 cells in response to SA stress (0.5 h, 0.2 mM SA) with or without one hour of recovery after the removal of SA. DAPI (blue) marks the nucleus, and mCherry-G3BP1 (red) marks SGs. Scale bars: 50 μm (WT and H1-3) and 20 μm (WT and H1-3 Zoom 4x). **(B)** Quantification of the percentage of HCT116 cells with SGs in (A); 95-154 cells were counted under each condition. **(C)** Quantification of the SG clearance rate calculated from (B). **(D)** Confocal images of WT or *C9orf72^-/-^* (KO-7 and KO-16) rat primary cerebral cortical neurons in response to SA stress (0.5 h, 0.05 mM SA) with or without one hour of recovery after the removal of SA. DAPI (blue) marks the nucleus, G3BP1 (green) marks SGs, and TUJ1 (red) marks neurons. Scale bars: 50 μm (G3BP1) and 20 μm (Zoom 4x G3BP1). **(E)** Quantification of the percentage of cerebral cortical neurons containing SGs in (D); 86-202 neurons were counted per condition. **(F)** Quantification of the SG clearance rate calculated from (E). **(G)** Immunofluorescence microscopy of HeLa cells expressing Flag-tagged proteins (Flag, Flag-C9S, Flag-C9T or Flag-C9L) treated with SA (0.2 mM) for 0.5 h with or without one hour of recovery after the removal of SA. DAPI (blue) marks the nucleus, G3BP1 (green) marks SGs, and Flag (red) stains Flag-tagged proteins. Scale bars: 50 μm. **(H)** Quantification of the percentage of cells containing SGs in HeLa cells expressing Flag-tagged proteins in (G); 92-175 cells were counted per condition. **(I)** Quantification of the SG clearance rate calculated from (H). **(J)** Lysates were prepared from HeLa cells overexpressing Flag-tagged proteins that were cultured under basal conditions, or SA stress conditions (0.5 h, 0.2 mM SA) with or without one hour of recovery. Flag-tagged proteins were immunoprecipitated with a Flag antibody pre-coupled to Protein A/G magnetic beads followed by western blot analysis using antibodies against Flag, eIF2α or p62. The asterisk (*) indicates a nonspecific band. In (B, C, E, F, H and I), data are presented as the means ± SD of three independent experiments. In (B, E, and H), two-way ANOVA was used and followed by Tukey's post hoc test. Unpaired two-tailed t-test was used in (C and F). In (I), one-way ANOVA was used, followed by Tukey's post hoc test. *P ≤ 0.05, **P ≤ 0.01, ***P ≤ 0.001, and ****P ≤ 0.0001.

Because the phosphorylation of eIF2α is a central trigger of SG formation 3, C9orf72 may be involved in SG formation by interacting with eIF2α or p-eIF2α (S51). In particular, under some conditions, both eIF2α and p-eIF2α (S51) are recruited to SGs 46, 47. In addition, the autophagy receptor p62 functions in the clearance of protein aggregates 48. A recent report shows that C9orf72 is in a complex with p62 and aids in eliminating SGs by autophagy 33. Considering these previously reported findings together, we infer that the loss of C9orf72 may influence the formation and/or removal of SGs. Here, we determined whether C9orf72 controlled the formation and/or clearance of SGs by treating HCT116 cells and primary cerebral cortical neurons with SA to induce SG formation, and G3BP1 was used as an SG marker. The percentage of *C9orf72^-/-^* cells with SGs increased significantly compared to that of WT cells after 0.5 h of SA treatment (Figure 6A, B, D, E). Additionally, after recovery from arsenite stress, the loss of C9orf72 suppressed SG elimination (Figure 6A-F). In summary, the loss of C9orf72 increases SG formation and impairs SG clearance. Furthermore, we reconstituted *C9orf72* KO HCT116 cells with Flag-tagged C9orf72 isoforms to assess the roles of C9orf72 isoforms in SG formation and clearance. Reconstitution with C9S or C9L, which interacted with eIF2α, reduced the levels of SG formation in *C9orf72* KO cells to those in WT cells (Figure S10A, B). Only reconstitution with C9L restored the SG clearance levels of *C9orf72* KO cells (Figure S10).

The presence of neuronal cytoplasmic aggregates containing the TAR DNA-binding protein 43 (TDP-43), which are considered SGs, is one of the pathological hallmarks of ALS and FTD 6. As the aforementioned data showed that C9orf72 played roles in SG formation and clearance, we attempted to investigate whether loss of C9orf72 led to the formation of cytoplasmic TDP-43 aggregates in the neurons of aged rats. However, cytoplasmic TDP-43 aggregates and TDP-43 mislocalization were not observed in the cerebral cortical neurons and spinal motor neurons of 17-month-old *C9orf72* KO rats (Figure S11), similar to the result from a conditional *C9orf72*-KO mouse model 49.

Finally, we overexpressed the Flag-tagged C9orf72 isoforms in HeLa cells to determine how C9orf72 affected SG formation and elimination by regulating the function of its interacting proteins eIF2α and p62. The overexpression of C9S or C9L in HeLa cells delayed SG formation under SA stress, but the overexpression of C9T in HeLa cells did not result in the same phenotype (Figure 6G, H). Additionally, only overexpression of C9L increased the clearance rate of SGs (Figure 6G-I). Finally, the co-IP studies showed that under basal conditions, both C9S and C9L tightly bound to eIF2α but that only C9L strongly interacted with p62 (Figure 6J). Compared with the control group, C9S and C9L showed stronger interactions with eIF2α when SGs formation and clearance were induced. Moreover, the level of p62 bound to C9L was increased under the conditions of SG formation and clearance (Figure 6J). These data indicate that the short isoform domain of C9orf72 is necessary for interaction with eIF2α and the regulation of SG formation. Only the full-length C9orf72 protein can bind p62 to help remove SGs in stressed cells.

## Discussion

In the present study, we explored how C9orf72 regulated the function of its interacting protein eIF2α in translation initiation and stress responses. Using HCT116 cell and rat models, we found that *C9orf72* KO repressed global translation by reducing the interaction of eIF2α with eIF2B5. Moreover, the loss of C9orf72 led to ER stress-induced inflammation in the spleen via the activation of the GRP78-IRE1-XBP1 axis and hyperformation and persistence of SGs in stressed cells. Defects in this process might be relevant early events in the initiation of C9ALS/FTD pathogenesis.

eIF2 is necessary for mRNA translation and can be regulated by phosphorylation at Ser51 of its eIF2α subunit. The evidence obtained in this study illustrates that C9orf72 interacts with eIF2α and p-eIF2α (S51) (Figure 2). The weakened interaction of eIF2α and eIF2B5 in *C9orf72^-/-^* HCT116 cell lines and rat tissues is assumed to be responsible for impairing the function of eIF2 in translation initiation, resulting in global translation inhibition in both unstressed and stressed cells (Figure 3). We propose that C9orf72 acts as a regulatory factor for the eIF2-eIF2B complex through its interaction with eIF2α. The eIF2-eIF2B interaction is mainly regulated by phosphorylation at Ser 51 of the eIF2α subunit 4. Several studies reporting the structures of unphosphorylated or phosphorylated eIF2 bound to eIF2B suggested that phosphorylation at Ser 51 of the eIF2α subunit may strengthen the eIF2-eIF2B interaction by changing the conformation of eIF2 or/and increasing the affinity for eIF2B 50-52. Moreover, when eIF2B catalyzes eIF2, the conformational change in eIF2 also alters the affinity of eIF2 and eIF2B 53. One possible mechanism by which the loss of the C9orf72 protein weakens the interaction of eIF2 with eIF2B is that the loss of C9orf72 alters the conformation of eIF2. Obtaining structural information for the C9orf72-eIF2 complex will definitely be the research focus of our future work. Dysfunction of eIF2B is associated with many neurodegenerative diseases. For example, many eIF2B mutations with reduced GEF activity cause leukoencephalopathy with vanishing white matter 54. Additionally, several reports show that ER stress and oxidative stress enhance RAN translation through the eIF2α-related integrated stress response 42, 55, 56. Therefore, some meaningful future studies will explore whether the loss of C9orf72 favors RAN translation by dysregulating the function of eIF2, creating a potential feed-forward loop that might contribute to the disease pathogenesis.

Unlike the gain-of-toxicity mechanism causing typical ALS-like motor deficits in mouse models 57-59, the *C9orf72*-null rats generated in this study did not develop motor deficits or anxiety-like behavior (Figure S2), similar to a *C9orf72*-KO mouse model 49. However, the *C9orf72*-null rats developed other distinct symptoms, such as visible splenomegaly and cervical lymphadenopathy (Figure 1C, D; Figure S1D, E). Strikingly, the expression of immune receptor genes (*Cxcr1* and *Trem2*) and inflammatory cytokine genes (*Ccl3*, *Ccl9* and *Il-1a*) was significantly increased in *C9orf72*-null rats (Figure 5A-C). These characteristics were reminiscent of age-related inflammation in the spleen. Several previous studies have revealed analogous features of *C9orf72*-deficient mice 34-36. Furthermore, the loss of *C9orf72* in mice caused microglial inflammation accompanied by lysosomal transport defects 35. The above findings suggest that people with low C9orf72 levels are susceptible to autoimmune diseases. In particular, epidemiologic studies have shown that some symptoms of autoimmune diseases precede ALS and FTD, leading to a greater risk of motor neuron destruction in patients with ALS/FTD 60, 61. Therefore, it is important to determine the upstream pathways of autoimmunity and inflammation triggered by the loss of C9orf72 and then to identify valuable upstream therapies that could prevent loss-of-*C9orf72*-linked neurological dysfunction.

A recent study showed that activation of the cGAS-STING pathway in *C9orf72*^-/-^ myeloid cells led to type I interferon-driven inflammation 37. Our work differs by showing that the activation of the GRP78-IRE1-XBP1 axis is one of the causes of splenomegaly and inflammation in *C9orf72*^-/-^ rats (Figure 4B; Figure 5D-F). Similarly, the IRE1-XBP1 branch activated by ER stress can act synergistically with Toll-like receptor (TLR) signaling to promote cytokine production in macrophages 62. Both* in vitro* and *in vivo* data have revealed the activation of the IRE1-XBP1 pathway in activated natural killer cells 63. Consequently, the IRE1-XBP1 pathway is involved in the process of immune responses. Moreover, the loss of XBP1 in intestinal epithelial cells results in increased ER stress-induced inflammation 64. TLRs activate XBP1 in macrophages to modulate innate immune responses 62. It is plausible that both activation and impairment of the IRE1-XBP1 axis lead to the dysregulation of the downstream inflammatory pathway. However, the biological link between the loss of *C9orf72* and GRP78-IRE1-XBP1 pathway-induced inflammation is still unclear, likely resulting from the cross-interaction among the three branches of the UPR. For instance, knockdown and pharmacological inhibition of PERK can enhance IRE1 phosphorylation under ER stress, and the expression of XBP1s or active ATF6 can upregulate the PERK-eIF2α branch 65, 66. Future investigations will test how the loss of C9orf72 activates the GRP78-IRE1-XBP1 axis and whether a similar altered pathway exists in C9ALS/FTD patients.

We further revealed that C9orf72 protected neurons from severe and prolonged ER stress (Figure S9). C9ALS/FTD patients with *C9orf72* haploinsufficiency might be more susceptible to stress-associated apoptosis. In addition, low C9orf72 expression in n2a cells and cortical neurons leads to sensitivity to cellular stress and strongly affects cell survival 24. With increasing age, people with reduced expression of C9orf72 are more likely to develop ALS/FTD because of an inability to protect neurons from aging-related stress. This could partly explain the pathogenic mechanism of *C9orf72* haploinsufficiency in C9ALS/FTD patients. Moreover, therapies aimed at alleviating stress in the brains and spinal cords of ALS/FTD patients may protect neurons from stress-associated apoptosis, thus delaying disease development.

Under stress stimuli, the assembly and disassembly of SGs are in dynamic balance with mRNA metabolism and protein translation 3. C9orf72, associated with p62, promotes the elimination of SGs via autophagy 33. In this study, we highlight the conclusion that the loss of C9orf72 promotes SG formation in cells under arsenite stress (Figure 6A, B, D, E), which is reminiscent of the role of UBQLN2 as a negative regulator of SG formation 67. However, knockdown of *C9orf72* does not increase SG formation in HeLa cells under arsenite stress 33. This discrepancy may be due to different *C9orf72* levels between knockout and knockdown and different cell lines. In addition, the reduced expression of VCP impairs SG clearance in mammalian cells 68. Together, these ALS-related proteins UBQLN2, VCP and C9orf72 are involved in the regulation of SG dynamics, indicating that aberrant SG dynamics underlie the pathogenesis of ALS. An interesting field of future research will be to understand how these proteins affect SG dynamics in molecular detail and the physiological consequences of defects in this process.

In fact, abnormal cytoplasmic inclusions containing TDP-43, which are considered SGs, are histologically detected in most ALS and FTD patients 6. Under stress conditions, SGs can assemble other ALS-related proteins, such as UBQLN2 and VCP 67, 68. ALS-causing mutations in UBQLN2 weaken its interaction with FUS, thus impairing the function of UBQLN2 in modulating SG formation 67. The ALS/FTD-linked mutation p62^G427R^ enhances the formation of TDP-43-positive SGs upon arsenite stress 69. Additionally, disease-linked mutations in VCP result in the formation of SG-containing TDP-43 and mutant VCP itself 68. Similarly, repetitive RNA and DPRs from GGGGCC repeat expansions of *C9orf72* induce the abnormal accumulation of SGs and disrupt the dynamics of SGs 70-74. Hence, these findings support the hypothesis that some related pathologies of ALS and FTD are caused by hyperformation or persistence of SGs. However, in certain circumstances, the overexpression of GGGGCC repeat expansions in mice does not reproduce all the pathological symptoms of ALS/FTD patients 59, 75. Notably, direct evidence suggests that the neurodegenerative features of patients with ALS/FTD are a joint consequence of loss-of-function and gain-of-toxicity mechanisms 23, 76, 77. The decrease in C9orf72 levels in neuron and mouse models disrupts endosome and lysosome functions as well as the autophagy pathway, which affects the removal of DPRs and thereby increases their cumulative toxicity, ultimately leading to neurodegeneration 23, 76, 77. Since C9orf72 is an effector of SG formation and elimination (Figure 6), we presume that *C9orf72* deficiency promotes pathological SG persistence caused by a gain of toxicity in patients with ALS/FTD, exacerbating the condition of patients. All of the above findings suggest that the loss of C9orf72 may act synergistically with a gain-of-toxicity mechanism in C9ALS/FTD pathogenesis and indicate that limiting SG hyperformation and enhancing SG removal may be feasible strategies for the treatment of C9ALS/FTD.

Collectively, our data show that C9orf72 is a molecular determinant implicated in the eIF2α-related stress response signaling pathway and thus plays roles in translation initiation, the development of ER stress-induced inflammation, the stress response and neuroprotection. Importantly, the environment-related gut microbiota significantly affects the phenotype of *C9orf72*-null mice, ultimately affecting their lifespan 78. These findings may explain the difference in lifespan between two groups of *C9orf72*-null mice with similar genetic backgrounds 34, 35, which emphasizes the importance of environmental factors in animal survival. Altogether, the evidence suggests that the loss of C9orf72, resulting in eIF2α-related stress response dysfunction and sensitivity of cells to the environment, may be involved in the full pathological spectrum of ALS and FTD.

## Supplementary Material

Supplementary figures and tables.

## References

[1] Hetz C (2012). The unfolded protein response: controlling cell fate decisions under ER stress and beyond. Nat Rev Mol Cell Biol.

[2] Dafinca R, Barbagallo P, Talbot K (2021). The Role of Mitochondrial Dysfunction and ER Stress in TDP-43 and C9ORF72 ALS. Front Cell Neurosci.

[3] Kedersha N, Ivanov P, Anderson P (2013). Stress granules and cell signaling: more than just a passing phase?. Trends Biochem Sci.

[4] Jackson RJ, Hellen CUT, Pestova TV (2010). The mechanism of eukaryotic translation initiation and principles of its regulation. Nat Rev Mol Cell Biol.

[5] Li YR, King OD, Shorter J, Gitler AD (2013). Stress granules as crucibles of ALS pathogenesis. J Cell Biol.

[6] Robberecht W, Philips T (2013). The changing scene of amyotrophic lateral sclerosis. Nat Rev Neurosci.

[7] Josephs KA, Hodges JR, Snowden JS, Mackenzie IR, Neumann M, Mann DM (2011). Neuropathological background of phenotypical variability in frontotemporal dementia. Acta Neuropathol.

[8] Dobson-Stone C, Hallupp M, Bartley L, Shepherd CE, Halliday GM, Schofield PR (2012). C9ORF72 repeat expansion in clinical and neuropathologic frontotemporal dementia cohorts. Neurology.

[9] Sha SJ, Takada LT, Rankin KP, Yokoyama JS, Rutherford NJ, Fong JC (2012). Frontotemporal dementia due to C9ORF72 mutations: clinical and imaging features. Neurology.

[10] DeJesus-Hernandez M, Mackenzie Ian R, Boeve Bradley F, Boxer Adam L, Baker M, Rutherford Nicola J (2011). Expanded GGGGCC hexanucleotide repeat in noncoding region of *C9ORF72* causes chromosome 9p-linked FTD and ALS. Neuron.

[11] Renton AE, Majounie E, Waite A, Simón-Sánchez J, Rollinson S, Gibbs JR (2011). A hexanucleotide repeat expansion in C9ORF72 is the cause of chromosome 9p21-linked ALS-FTD. Neuron.

[12] Majounie E, Renton AE, Mok K, Dopper EGP, Waite A, Rollinson S (2012). Frequency of the *C9orf72* hexanucleotide repeat expansion in patients with amyotrophic lateral sclerosis and frontotemporal dementia: a cross-sectional study. Lancet Neurol.

[13] Donnelly CJ, Zhang P-W, Pham JT, Haeusler AR, Mistry NA, Vidensky S (2013). RNA toxicity from the ALS/FTD C9ORF72 expansion is mitigated by antisense intervention. Neuron.

[14] Gendron TF, Bieniek KF, Zhang Y-J, Jansen-West K, Ash PEA, Caulfield T (2013). Antisense transcripts of the expanded C9ORF72 hexanucleotide repeat form nuclear RNA foci and undergo repeat-associated non-ATG translation in c9FTD/ALS. Acta Neuropathol.

[15] Zu T, Liu Y, Bañez-Coronel M, Reid T, Pletnikova O, Lewis J (2013). RAN proteins and RNA foci from antisense transcripts in *C9ORF72* ALS and frontotemporal dementia. Proc Natl Acad Sci U S A.

[16] Ash PEA, Bieniek KF, Gendron TF, Caulfield T, Lin W-L, Dejesus-Hernandez M (2013). Unconventional translation of C9ORF72 GGGGCC expansion generates insoluble polypeptides specific to c9FTD/ALS. Neuron.

[17] Mori K, Weng S-M, Arzberger T, May S, Rentzsch K, Kremmer E (2013). The *C9orf72* GGGGCC repeat is translated into aggregating dipeptide-repeat proteins in FTLD/ALS. Science.

[18] Gendron TF, van Blitterswijk M, Bieniek KF, Daughrity LM, Jiang J, Rush BK (2015). Cerebellar c9RAN proteins associate with clinical and neuropathological characteristics of C9ORF72 repeat expansion carriers. Acta Neuropathol.

[19] Saberi S, Stauffer JE, Jiang J, Garcia SD, Taylor AE, Schulte D (2018). Sense-encoded poly-GR dipeptide repeat proteins correlate to neurodegeneration and uniquely co-localize with TDP-43 in dendrites of repeat-expanded C9orf72 amyotrophic lateral sclerosis. Acta Neuropathol.

[20] Gijselinck I, Van Langenhove T, van der Zee J, Sleegers K, Philtjens S, Kleinberger G (2012). A C9orf72 promoter repeat expansion in a Flanders-Belgian cohort with disorders of the frontotemporal lobar degeneration-amyotrophic lateral sclerosis spectrum: a gene identification study. Lancet Neurol.

[21] Belzil VV, Bauer PO, Prudencio M, Gendron TF, Stetler CT, Yan IK (2013). Reduced *C9orf72* gene expression in c9FTD/ALS is caused by histone trimethylation, an epigenetic event detectable in blood. Acta Neuropathol.

[22] van Blitterswijk M, Gendron TF, Baker MC, DeJesus-Hernandez M, Finch NA, Brown PH (2015). Novel clinical associations with specific C9ORF72 transcripts in patients with repeat expansions in C9ORF72. Acta Neuropathol.

[23] Shi Y, Lin S, Staats KA, Li Y, Chang W-H, Hung S-T (2018). Haploinsufficiency leads to neurodegeneration in C9ORF72 ALS/FTD human induced motor neurons. Nat Med.

[24] Maharjan N, Künzli C, Buthey K, Saxena S (2017). C9orf72 regulates stress granule formation and its deficiency impairs stress granule assembly, hypersensitizing cells to stress. Mol Neurobiol.

[25] Xiao S, MacNair L, McGoldrick P, McKeever PM, McLean JR, Zhang M (2015). Isoform-specific antibodies reveal distinct subcellular localizations of C9orf72 in amyotrophic lateral sclerosis. Ann Neurol.

[26] Zhang D, Iyer L, He F, Aravind L (2012). Discovery of novel DENN proteins: implications for the evolution of eukaryotic intracellular membrane structures and human disease. Front Genet.

[27] Levine TP, Daniels RD, Gatta AT, Wong LH, Hayes MJ (2013). The product of C9orf72, a gene strongly implicated in neurodegeneration, is structurally related to DENN Rab-GEFs. Bioinformatics.

[28] Sellier C, Campanari M-L, Julie Corbier C, Gaucherot A, Kolb-Cheynel I, Oulad-Abdelghani M (2016). Loss of C9ORF72 impairs autophagy and synergizes with polyQ Ataxin-2 to induce motor neuron dysfunction and cell death. EMBO J.

[29] Webster CP, Smith EF, Bauer CS, Moller A, Hautbergue GM, Ferraiuolo L (2016). The C9orf72 protein interacts with Rab1a and the ULK1 complex to regulate initiation of autophagy. EMBO J.

[30] Yang M, Liang C, Swaminathan K, Herrlinger S, Lai F, Shiekhattar R (2016). A C9ORF72/SMCR8-containing complex regulates ULK1 and plays a dual role in autophagy. Sci Adv.

[31] Aoki Y, Manzano R, Lee Y, Dafinca R, Aoki M, Douglas AGL (2017). C9orf72 and RAB7L1 regulate vesicle trafficking in amyotrophic lateral sclerosis and frontotemporal dementia. Brain.

[32] Corrionero A, Horvitz HR (2018). A C9ORF72 ALS/FTD ortholog acts in endolysosomal degradation and lysosomal homeostasis. Curr Biol.

[33] Chitiprolu M, Jagow C, Tremblay V, Bondy-Chorney E, Paris G, Savard A (2018). A complex of C9ORF72 and p62 uses arginine methylation to eliminate stress granules by autophagy. Nat Commun.

[34] Burberry A, Suzuki N, Wang J-Y, Moccia R, Mordes DA, Stewart MH (2016). Loss-of-function mutations in the *C9ORF72* mouse ortholog cause fatal autoimmune disease. Sci Transl Med.

[35] O'Rourke JG, Bogdanik L, Yáñez A, Lall D, Wolf AJ, Muhammad AKMG (2016). *C9orf72* is required for proper macrophage and microglial function in mice. Science.

[36] Atanasio A, Decman V, White D, Ramos M, Ikiz B, Lee H-C (2016). *C9orf72* ablation causes immune dysregulation characterized by leukocyte expansion, autoantibody production and glomerulonephropathy in mice. Sci Rep.

[37] McCauley ME, O'Rourke JG, Yáñez A, Markman JL, Ho R, Wang X (2020). C9orf72 in myeloid cells suppresses STING-induced inflammation. Nature.

[38] Pacifici M, Peruzzi F Isolation and culture of rat embryonic neural cells: a quick protocol. J Vis Exp. 2012: e3965.

[39] Schmidt EK, Clavarino G, Ceppi M, Pierre P (2009). SUnSET, a nonradioactive method to monitor protein synthesis. Nat Methods.

[40] Jiang D, Zou X, Zhang C, Chen J, Li Z, Wang Y (2018). Gemin5 plays a role in unassembled-U1 snRNA disposal in SMN-deficient cells. FEBS Lett.

[41] Gomez E, Pavitt GD (2000). Identification of domains and residues within the ɛ subunit of eukaryotic translation initiation factor 2b (eIF2Bɛ) required for guanine nucleotide exchange reveals a novel activation function promoted by eIF2B complex formation. Mol Cell Biol.

[42] Green KM, Glineburg MR, Kearse MG, Flores BN, Linsalata AE, Fedak SJ (2017). RAN translation at *C9orf72*-associated repeat expansions is selectively enhanced by the integrated stress response. Nat Commun.

[43] Hallek M, Shanafelt TD, Eichhorst B (2018). Chronic lymphocytic leukaemia. Lancet.

[44] Grootjans J, Kaser A, Kaufman RJ, Blumberg RS (2016). The unfolded protein response in immunity and inflammation. Nat Rev Immunol.

[45] Deng H, Kuang P, Cui H, Chen L, Luo Q, Fang J (2016). Sodium fluoride (NaF) induces the splenic apoptosis via endoplasmic reticulum (ER) stress pathway *in vivo* and *in vitro*. Aging.

[46] Kedersha N, Chen S, Gilks N, Li W, Miller IJ, Stahl J (2002). Evidence that ternary complex (eIF2-GTP-tRNA_i_^Met^)-deficient preinitiation complexes are core constituents of mammalian stress granules. Mol Biol Cell.

[47] Kimball SR, Horetsky RL, Ron D, Jefferson LS, Harding HP (2003). Mammalian stress granules represent sites of accumulation of stalled translation initiation complexes. Am J Physiol Cell Physiol.

[48] Deng Z, Purtell K, Lachance V, Wold MS, Chen S, Yue Z (2017). Autophagy receptors and neurodegenerative diseases. Trends Cell Biol.

[49] Koppers M, Blokhuis AM, Westeneng H-J, Terpstra ML, Zundel CAC, Vieira de Sá R (2015). C9orf72 ablation in mice does not cause motor neuron degeneration or motor deficits. Ann Neurol.

[50] Kenner LR, Anand AA, Nguyen HC, Myasnikov AG, Klose CJ, McGeever LA (2019). eIF2B-catalyzed nucleotide exchange and phosphoregulation by the integrated stress response. Science.

[51] Kashiwagi K, Yokoyama T, Nishimoto M, Takahashi M, Sakamoto A, Yonemochi M (2019). Structural basis for eIF2B inhibition in integrated stress response. Science.

[52] Adomavicius T, Guaita M, Zhou Y, Jennings MD, Latif Z, Roseman AM (2019). The structural basis of translational control by eIF2 phosphorylation. Nat Commun.

[53] Bogorad AM, Lin KY, Marintchev A (2017). Novel mechanisms of eIF2B action and regulation by eIF2α phosphorylation. Nucleic Acids Res.

[54] Fogli A, Schiffmann R, Hugendubler L, Combes P, Bertini E, Rodriguez D (2004). Decreased guanine nucleotide exchange factor activity in eIF2B-mutated patients. Eur J Hum Genet.

[55] Sonobe Y, Ghadge G, Masaki K, Sendoel A, Fuchs E, Roos RP (2018). Translation of dipeptide repeat proteins from the C9ORF72 expanded repeat is associated with cellular stress. Neurobiol Dis.

[56] Cheng W, Wang S, Mestre AA, Fu C, Makarem A, Xian F (2018). C9ORF72 GGGGCC repeat-associated non-AUG translation is upregulated by stress through eIF2α phosphorylation. Nat Commun.

[57] Chew J, Gendron TF, Prudencio M, Sasaguri H, Zhang Y-J, Castanedes-Casey M (2015). *C9ORF72* repeat expansions in mice cause TDP-43 pathology, neuronal loss, and behavioral deficits. Science.

[58] Liu Y, Pattamatta A, Zu T, Reid T, Bardhi O, Borchelt David R (2016). *C9orf72* BAC mouse model with motor deficits and neurodegenerative features of ALS/FTD. Neuron.

[59] Schludi MH, Becker L, Garrett L, Gendron TF, Zhou Q, Schreiber F (2017). Spinal poly-GA inclusions in a *C9orf72* mouse model trigger motor deficits and inflammation without neuron loss. Acta Neuropathol.

[60] Miller ZA, Sturm VE, Camsari GB, Karydas A, Yokoyama JS, Grinberg LT (2016). Increased prevalence of autoimmune disease within C9 and FTD/MND cohorts: completing the picture. Neurol Neuroimmunol Neuroinflamm.

[61] Turner MR, Goldacre R, Ramagopalan S, Talbot K, Goldacre MJ (2013). Autoimmune disease preceding amyotrophic lateral sclerosis: an epidemiologic study. Neurology.

[62] Martinon F, Chen X, Lee A-H, Glimcher LH (2010). TLR activation of the transcription factor XBP1 regulates innate immune responses in macrophages. Nat Immunol.

[63] Dong H, Adams NM, Xu Y, Cao J, Allan DSJ, Carlyle JR (2019). The IRE1 endoplasmic reticulum stress sensor activates natural killer cell immunity in part by regulating c-Myc. Nat Immunol.

[64] Kaser A, Lee A-H, Franke A, Glickman JN, Zeissig S, Tilg H (2008). Xbp1 links ER stress to intestinal inflammation and confers genetic risk for human inflammatory bowel disease. Cell.

[65] Chang T-K, Lawrence DA, Lu M, Tan J, Harnoss JM, Marsters SA (2018). Coordination between two branches of the unfolded protein response determines apoptotic cell fate. Mol Cell.

[66] Spaan CN, Smit WL, van Lidth de Jeude JF, Meijer BJ, Muncan V, van den Brink GR (2019). Expression of UPR effector proteins ATF6 and XBP1 reduce colorectal cancer cell proliferation and stemness by activating PERK signaling. Cell Death Dis.

[67] Alexander EJ, Ghanbari Niaki A, Zhang T, Sarkar J, Liu Y, Nirujogi RS (2018). Ubiquilin 2 modulates ALS/FTD-linked FUS-RNA complex dynamics and stress granule formation. Proc Natl Acad Sci U S A.

[68] Buchan JR, Kolaitis R-M, Taylor JP, Parker R (2013). Eukaryotic stress granules are cleared by autophagy and Cdc48/VCP function. Cell.

[69] Deng Z, Lim J, Wang Q, Purtell K, Wu S, Palomo GM (2020). ALS-FTLD-linked mutations of SQSTM1/p62 disrupt selective autophagy and NFE2L2/NRF2 anti-oxidative stress pathway. Autophagy.

[70] Hartmann H, Hornburg D, Czuppa M, Bader J, Michaelsen M, Farny D (2018). Proteomics and *C9orf72* neuropathology identify ribosomes as poly-GR/PR interactors driving toxicity. Life Sci Alliance.

[71] Lee K-H, Zhang P, Kim HJ, Mitrea DM, Sarkar M, Freibaum BD (2016). C9orf72 dipeptide repeats impair the assembly, dynamics, and function of membrane-less organelles. Cell.

[72] Chew J, Cook C, Gendron TF, Jansen-West K, del Rosso G, Daughrity LM (2019). Aberrant deposition of stress granule-resident proteins linked to *C9orf72*-associated TDP-43 proteinopathy. Mol Neurodegener.

[73] Boeynaems S, Bogaert E, Kovacs D, Konijnenberg A, Timmerman E, Volkov A (2017). Phase separation of *C9orf72* dipeptide repeats perturbs stress granule dynamics. Mol Cell.

[74] Fay MM, Anderson PJ, Ivanov P (2017). ALS/FTD-associated C9ORF72 repeat RNA promotes phase transitions *in vitro* and in cells. Cell Rep.

[75] Peters OM, Cabrera GT, Tran H, Gendron TF, McKeon JE, Metterville J (2015). Human *C9ORF72* hexanucleotide expansion reproduces RNA foci and dipeptide repeat proteins but not neurodegeneration in BAC transgenic mice. Neuron.

[76] Boivin M, Pfister V, Gaucherot A, Ruffenach F, Negroni L, Sellier C (2020). Reduced autophagy upon C9ORF72 loss synergizes with dipeptide repeat protein toxicity in G4C2 repeat expansion disorders. EMBO J.

[77] Zhu Q, Jiang J, Gendron TF, McAlonis-Downes M, Jiang L, Taylor A (2020). Reduced C9ORF72 function exacerbates gain of toxicity from ALS/FTD-causing repeat expansion in *C9orf72*. Nat Neurosci.

[78] Burberry A, Wells MF, Limone F, Couto A, Smith KS, Keaney J (2020). *C9orf72* suppresses systemic and neural inflammation induced by gut bacteria. Nature.

