# Supplementary material for: C9orf72 regulates the unfolded protein response and stress granule formation by interacting with eIF2α
Source: Theranostics. 2022 Oct 17;12(17):7289–306. doi: 10.7150/thno.76138 (PMC9691347; doi:10.7150/thno.76138)
Supplement: Supplementary file 1 — Supplementary figures and tables. [file thnov12p7289s1.pdf]

Supplementary material for:

C9orf72 regulates the unfolded protein response and stress granule formation by interacting with eIF2α

Wenzhong Zheng[1#], Kexin Wang[1#], Yachen Wu[1#], Ge Yan[1,3], Chi Zhang[1,3], Zhiqiang Li[1], Lianrong Wang[1,2*] & Shi Chen[1,3*]

#These authors contributed equally

*For correspondence: Professor Shi Chen, shichen_2021@163.com; Professor Lianrong Wang, lianrong@whu.edu.cn

This PDF file includes:

Figures S1 to S11

Tables S1 and S2

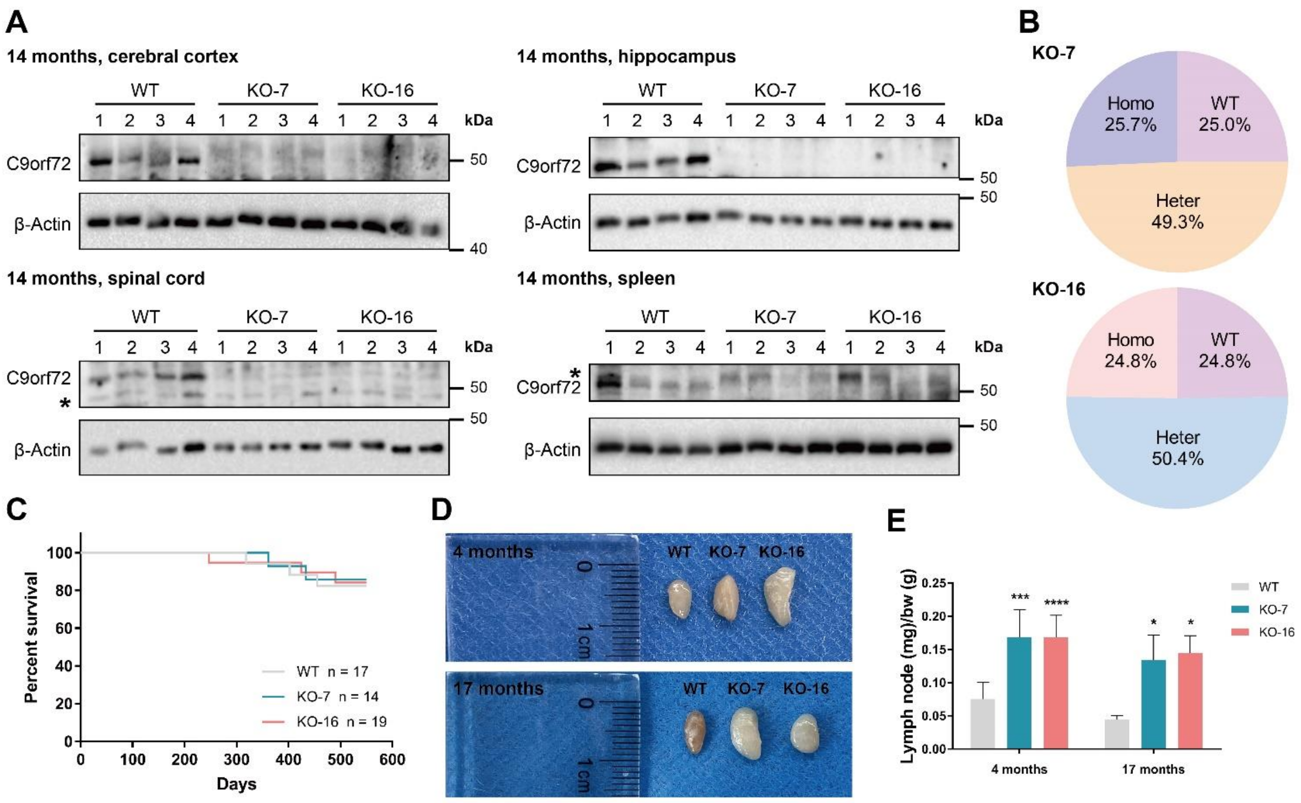

Figure S1. Gross characterization of *C9orf72*-KO rats. Related to Figure 1.

(A) Western blot analyses of C9orf72 protein levels in the cerebral cortex, hippocampus, spinal cord and spleen of 14-month-old WT and *C9orf72*-KO (KO-7 and KO-16) rats, with β-Actin serving as the loading control. The C9orf72 antibody (customized by GenScript) detected a band at 50 kDa that was not present in the KO-7 and KO-16 lysates. The asterisk (*) indicates a nonspecific band. **(B)** Crossing of heterozygous rats produced all genotypes (wildtype, heterozygous and homozygous KO pups) in the expected Mendelian ratio (1:2:1). **(C)** The survival curve of *C9orf72*-KO rats did not differ from WT rats up to 550 days. **(D)** Representative images of cervical lymph nodes from WT and *C9orf72*-KO rats at the indicated ages. **(E)** Cervical lymph node weights (in milligrams) normalized to body weight (in grams) at the indicated ages (n = 8 rats of each genotype at the age of 4 months, n = 3

rats of each genotype at the age of 17 months, means ± SD, unpaired two-tailed t-test, *$P \leq 0.05$, ***$P \leq 0.001$, and ****$P \leq 0.0001$).

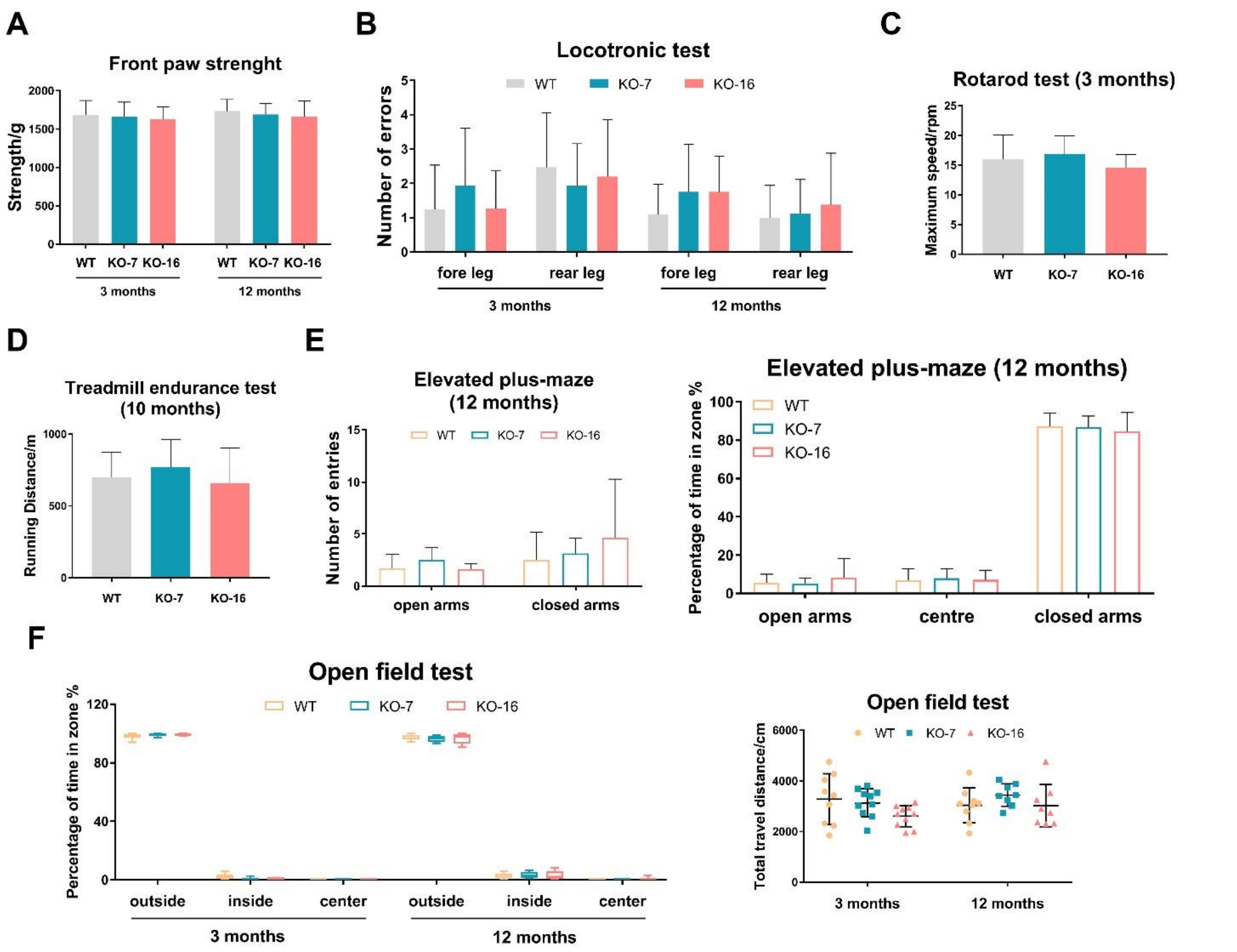

Figure S2. *C9orf72*-null rats do not develop motor deficits or anxiety-like behavior.

(A) Front paw strength of WT and *C9orf72*-null (KO-7 and KO-16) rats at 3 months and 12 months. **(B)** Number of errors in WT and *C9orf72*-null (KO-7 and KO-16) rats at 3 months and 12 months in the locotronic test. **(C)** Maximum rotarod speed of 3-month-old WT and *C9orf72*-null (KO-7 and KO-16) rats. **(D)** Running distance of 10-month-old WT and *C9orf72*-null (KO-7 and KO-16) rats in the treadmill endurance test. **(E)** Number of entries into open arms and closed arms (left panel) and percentages of time spent in the three regions (right panel) among 12-month-old WT and *C9orf72*-null (KO-7 and KO-16) rats in the elevated plus-

maze test. **(F)** Percentage of time spent in the three regions (left panel) and total traveling distance (right panel) among WT and *C9orf72*-null (KO-7 and KO-16) rats at 3 months and 12 months in the open field test. In (A-F), data are presented as means ± SD (n ≥ 8 rats of each genotype at each time point, unpaired two-tailed t-test).

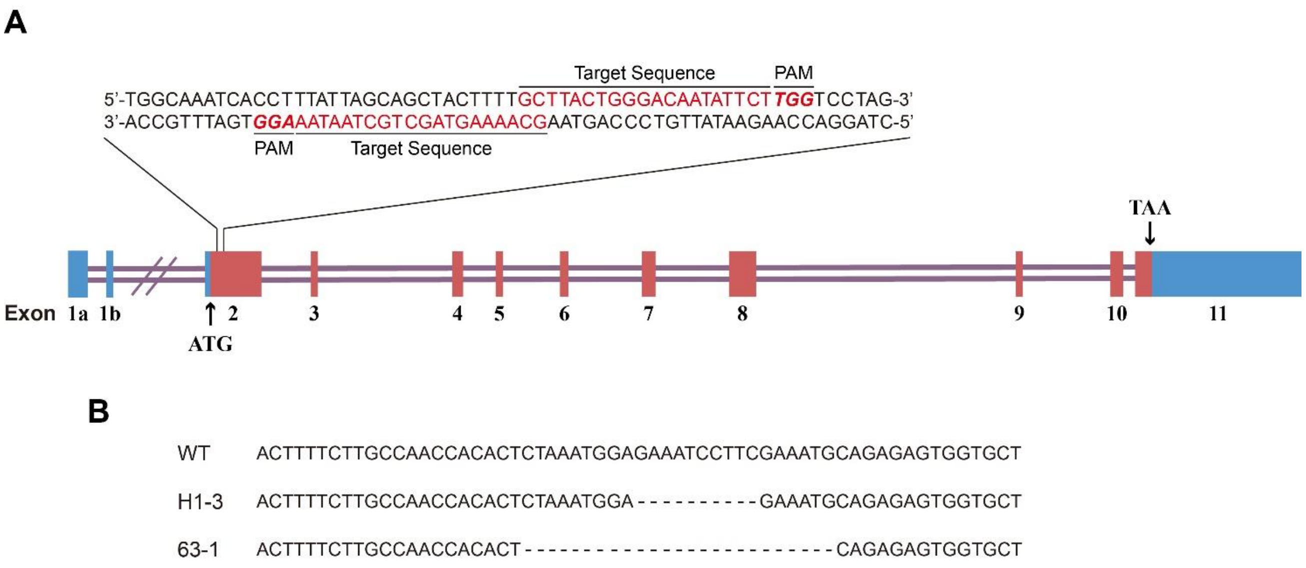

Figure S3. CRISPR/Cas9 gene editing technology was used to knock out *C9orf72* in HCT116 cells.

(A) Schematic of the human *C9orf72* gene structure, and the targeted sequences for CRISPR/Cas9 editing (the underlined sequences). The protospacer adjacent motif (PAM) sequences are indicated by italicized and bolded letters. **(B)** Alignment of sequences from the WT and *C9orf72*-KO (H1-3 and 63-1) cell lines. The deleted sequences in the *C9orf72*-KO (H1-3 and 63-1) cell lines were located downstream of the Cas9 cleavage sites.

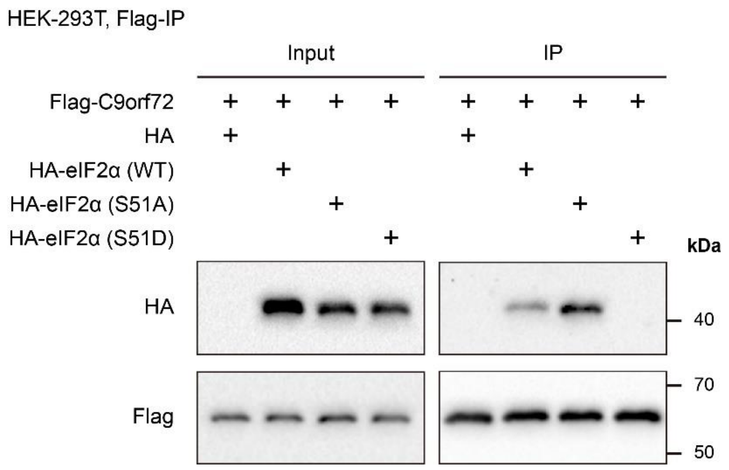

Figure S4. The levels of the nonphosphorylatable eIF2α (S51A) bound to C9orf72 were higher than those of the phosphomimetic eIF2α (S51D) bound to C9orf72. Related to Figure 2.

Flag-C9orf72 (long isoform) was co-expressed with the indicated HA-tagged protein in HEK-293T cells and IP was performed using Flag antibody pre-coupled to Protein A/G magnetic beads, followed by western blot analysis using antibodies against Flag or HA. eIF2α (WT), wild-type eIF2α; eIF2α (S51A), the nonphosphorylatable form of eIF2α; eIF2α (S51D), the phosphomimetic form of eIF2α.

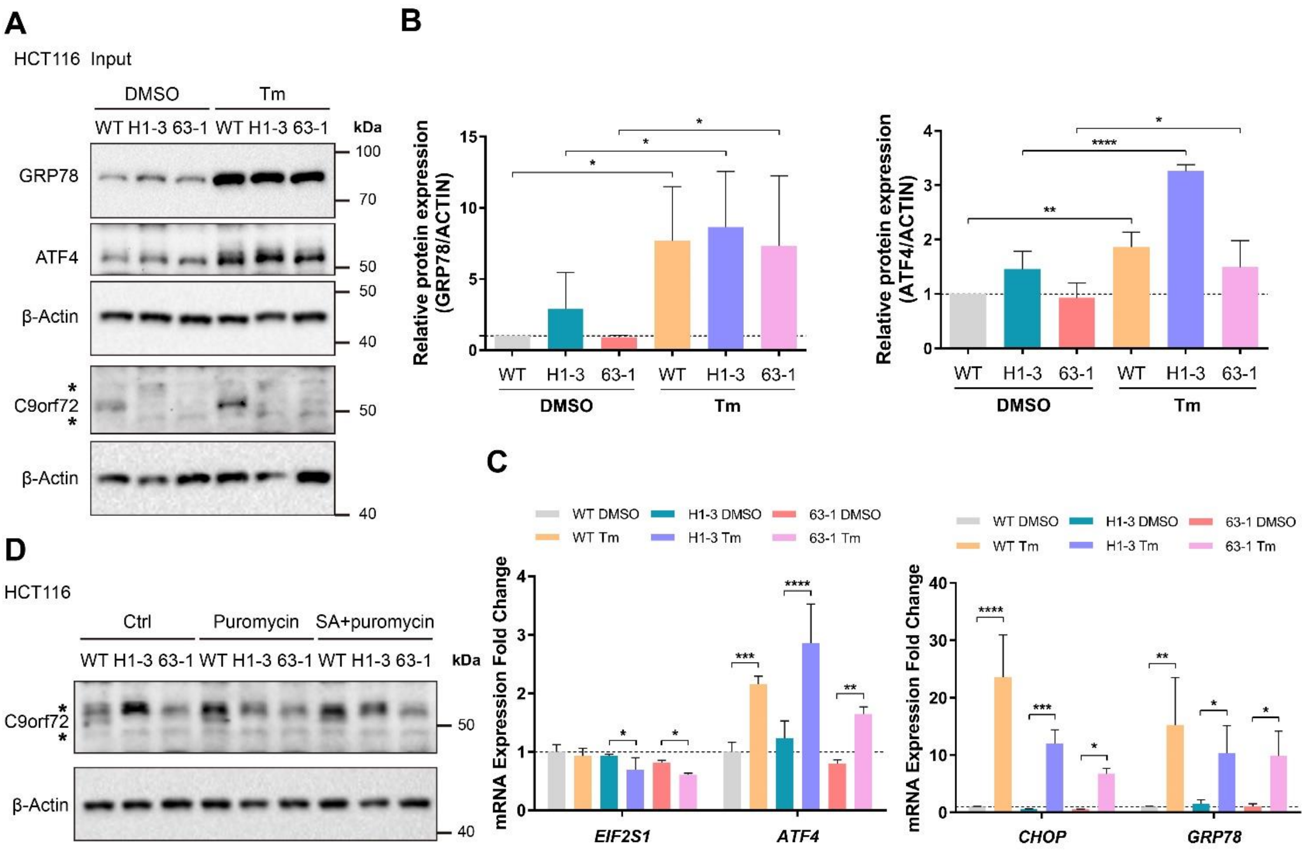

Figure S5. Tm induces ER stress in HCT116 cells. Related to Figure 3.

(A) Western blot analysis of GRP78 and ATF4 levels in either the DMSO- or Tm (1 μg/mL for 24 h)-treated WT and *C9orf72*$^{-/-}$ (H1-3 and 63-1) HCT116 cell lines (the input samples were obtained from the endogenous eIF2α IP assays shown in Figure 3A). The loss of the C9orf72 protein in *C9orf72*$^{-/-}$ cell lines (H1-3 and 63-1) was detected by western blotting with an anti-C9orf72 antibody (customized by GenScript). **(B)** Densitometry quantification of GRP78 (left panel) and ATF4 (right panel) levels based on the western blot results (n = 3 independent experiments, means ± SD, two-way ANOVA with Fisher's LSD test, *$P \leq 0.05$, **$P \leq 0.01$, and ****$P \leq 0.0001$). **(C)** RT-qPCR assay of *EIF2S1*, *ATF4*, *CHOP*, and *GRP78* mRNA expression

in WT, H1-3 and 63-1 cell lines treated with either vehicle (DMSO) or Tm (n = 3 independent experiments, means ± SD, two-way ANOVA with Fisher's LSD test, **P* ≤ 0.05, ***P* ≤ 0.01, ****P* ≤ 0.001, and *****P* ≤ 0.0001). **(D)** Western blot analysis of C9orf72 protein levels in the Ctrl group (untreated HCT116 cell lines, negative control), Puromycin group (HCT116 cell lines treated with 3 μg/mL puromycin for 30 min) and SA + puromycin group (HCT116 cell lines cotreated with 0.2 mM SA and 3 μg/mL puromycin for 30 min). β-Actin was used as a loading control. The asterisk (*) indicates a nonspecific band.

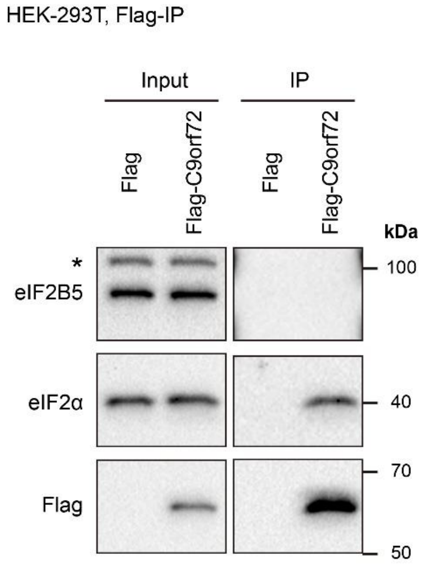

Figure S6. eIF2B5 is not pulled down together with eIF2α when immunoprecipitating Flag-C9orf72. Related to Figure 3.

Lysates were prepared from HEK-293T cells overexpressing Flag-tag or Flag-C9orf72 (long isoform). Flag-tagged proteins were immunoprecipitated with a Flag antibody pre-coupled to Protein A/G magnetic beads, followed by western blot analyses using antibodies against Flag, eIF2α or eIF2B5.

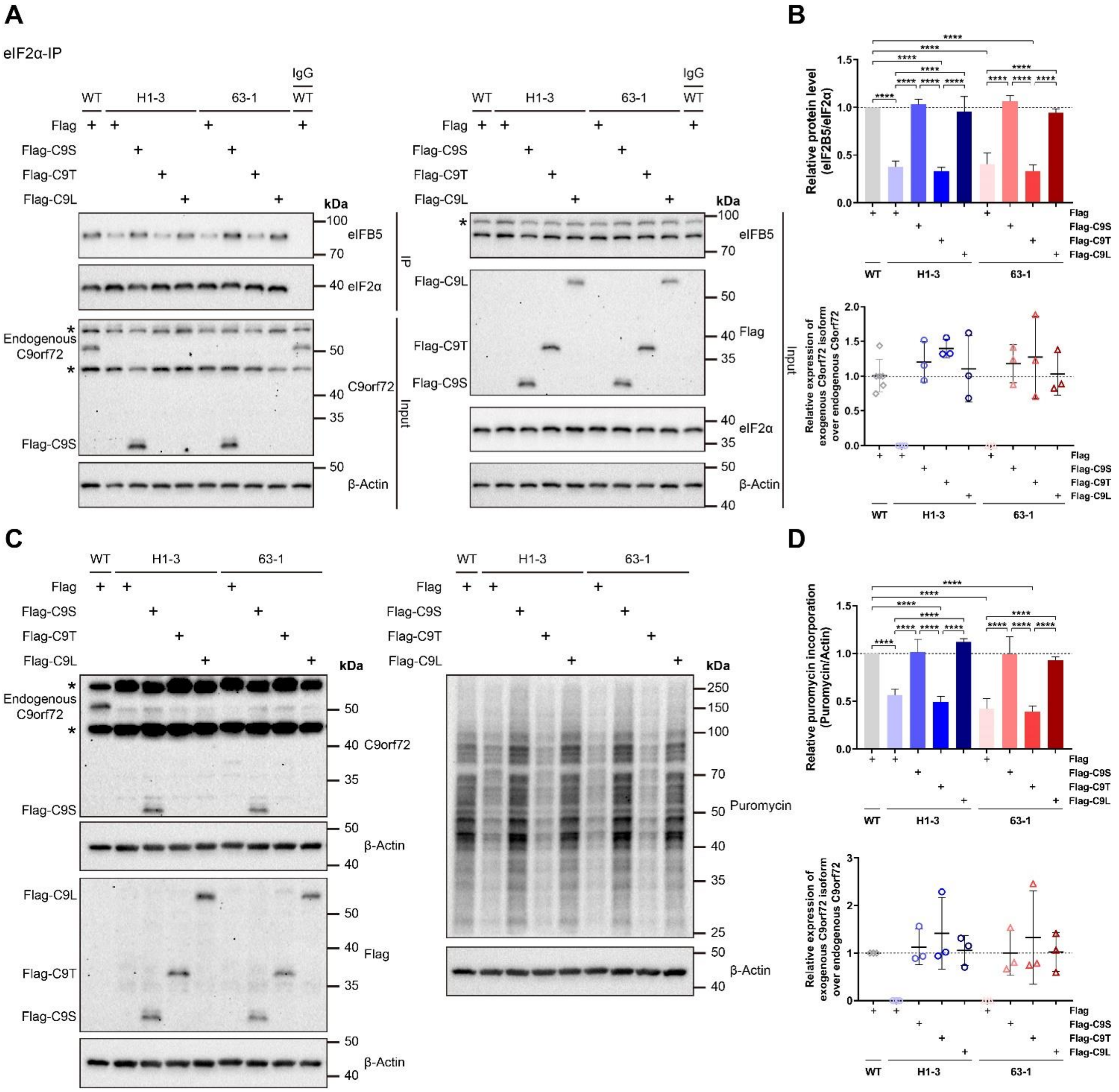

Figure S7. Reconstitution with C9S or C9L restores the interaction of eIF2α with eIF2B5 and the global translation levels of *C9orf72*$^{-/-}$ HCT116 cells. Related to Figure 3.

(A) Lysates were prepared from WT or *C9orf72*$^{-/-}$ (H1-3 and 63-1) HCT116 cells expressing the indicated Flag-tagged proteins. Endogenous eIF2α was immunoprecipitated with an eIF2α antibody pre-coupled to Protein A/G magnetic beads followed by western blot analyses

using antibodies against eIF2α or eIF2B5. The levels of endogenous C9orf72 and exogenous Flag-tag proteins in the input samples were also determined using western blotting. The asterisk (*) indicates a nonspecific band. **(B)** Relative ratio of eIF2B5 to eIF2α (upper panel) and relative ratio of the exogenous Flag-tag C9orf72 isoform to endogenous C9orf72 (lower panel) based on the western blot results (A) (n = 3 independent experiments, means ± SD, two-way ANOVA and followed by Tukey's post hoc test, ****$P \leq 0.0001$). **(C)** WT and *C9orf72*$^{-/-}$ (H1-3 and 63-1) HCT116 cells were transfected with the indicated plasmids. The cells were treated with 3 μg/mL puromycin for 30 min. Then, puromycin incorporation and the levels of endogenous C9orf72 and exogenous Flag-tag proteins were determined using western blotting. The asterisk (*) indicates a nonspecific band. **(D)** Quantification of puromycin incorporation (upper panel) and the relative ratio of exogenous Flag-tag C9orf72 isoform to endogenous C9orf72 (lower panel) based on the western blot results (C) (n = 3 independent experiments, means ± SD, two-way ANOVA and followed by Tukey's post hoc test, ****$P \leq 0.0001$).

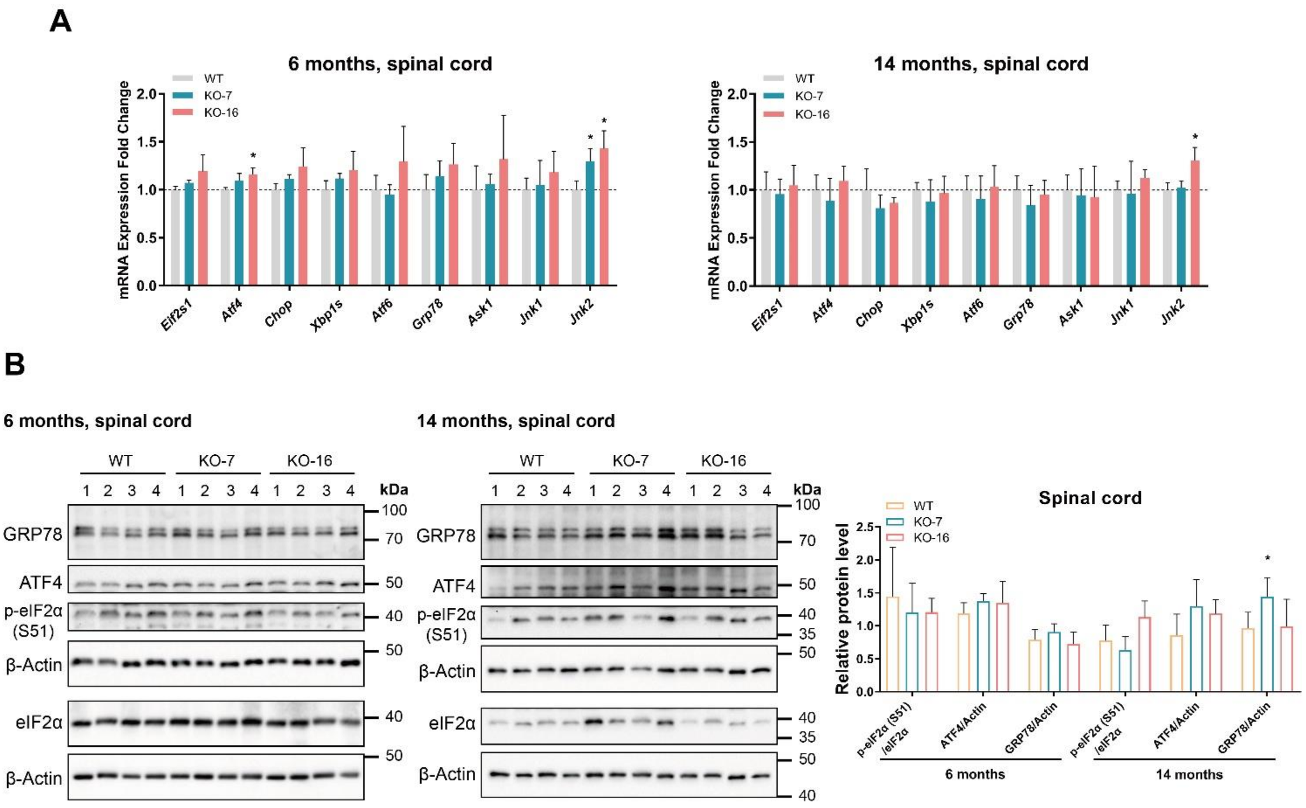

Figure S8. Loss of C9orf72 does not induce ER stress in the rat spinal cord. Related to Figure 4.

(A) Analyses of the mRNA expression of genes involved in ER stress pathways in the spinal cord from 6-month-old (left panel) and 14-month-old (right panel) male WT, KO-7 and KO-16 rats (n = 3 rats of each genotype, means ± SD, unpaired two-tailed t-test, *$P \leq 0.05$). **(B)** Western blot analyses of ER stress marker (the ratio of p-eIF2α (S51) to eIF2α, ATF4 and GRP78) levels in the spinal cords of 6-month-old (left panel) and 14-month-old (middle panel) male WT, KO-7 and KO-16 rats. The quantification of western blot results is shown in the charts on the right (n = 4 rats of each genotype, means ± SD, unpaired two-tailed t-test, *$P \leq 0.05$). β-Actin was used as a loading control.

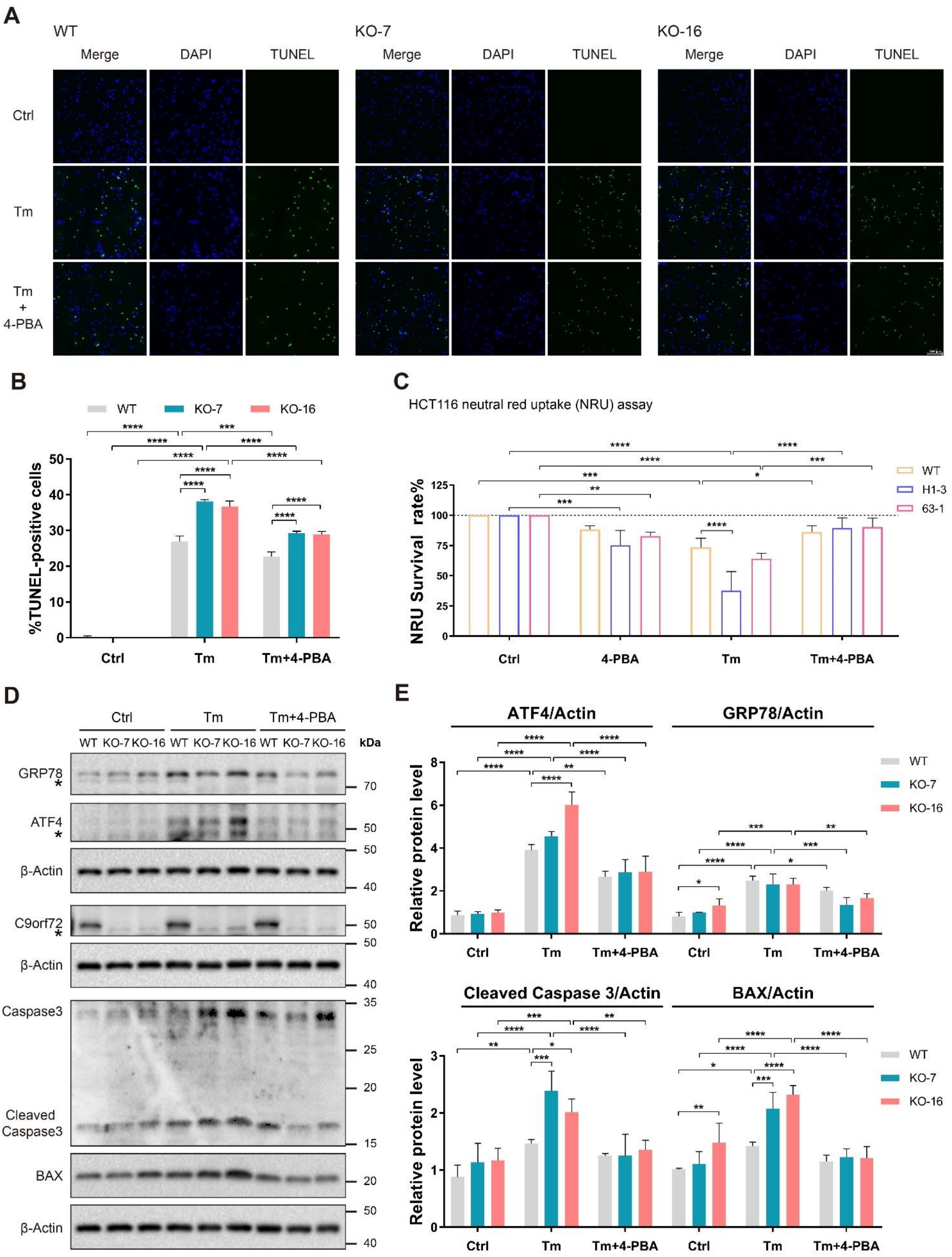
A
WT
KO-7
KO-16
Merge
DAPI
TUNEL
Ctrl
Tm
Tm + 4-PBA
B
WT
KO-7
KO-16
%TUNEL-positive cells
Ctrl
Tm
Tm+4-PBA
C
HCT116 neutral red uptake (NRU) assay
NRU Survival rate%
WT
H1-3
63-1
Ctrl
4-PBA
Tm
Tm+4-PBA
D
Ctrl
Tm
Tm+4-PBA
WT KO-7 KO-16 WT KO-7 KO-16 WT KO-7 KO-16
kDa
GRP78
ATF4
β-Actin
C9orf72
Caspase3
Cleaved Caspase3
BAX
E
ATF4/Actin
GRP78/Actin
Cleaved Caspase 3/Actin
BAX/Actin
Relative protein level
WT
KO-7
KO-16
Ctrl
Tm
Tm+4-PBA

Figure S9. ER stress induced by Tm increases apoptosis in *C9orf72*-null rat primary cerebral cortical neurons and HCT116 cells.

(A) TUNEL staining of 10-day primary rat cerebral cortical neurons treated with Tm (5 μg/mL for 12 h) and/or 4-PBA (8 mM for 24 h). All cells are stained blue with DAPI, and apoptotic cells are stained green with TUNEL. Scale bars: 100 μm. **(B)** Quantification of the percentage of TUNEL-positive cells in (A); 924–2439 cells were counted under each condition. (n = 3 independent experiments, means ± SD, two-way ANOVA and followed by Tukey's post hoc test, ***$P \leq 0.001$ and ****$P \leq 0.0001$). **(C)** HCT116 cell survival rate in the NRU assay. Cells were incubated with Tm (1 μg/mL, 16 h) and/or 4-PBA (3 mM, 24 h) (n = 3 independent experiments, means ± SD, two-way ANOVA with Fisher's LSD test, *$P \leq 0.05$, **$P \leq 0.01$, ***$P \leq 0.001$, and ****$P \leq 0.0001$). **(D)** GRP78, ATF4, C9orf72, cleaved Caspase3 and BAX expression levels were analyzed using western blotting in 10-day primary rat cerebral cortical neurons treated with Tm (5 μg/mL for 12 h) and/or 4-PBA (8 mM for 24 h). β-Actin was used as an internal reference. **(E)** Relative levels of ATF4, GRP78, cleaved Caspase3 and BAX based on the western blot results (D) (n = 3 independent experiments, means ± SD, two-way ANOVA with Fisher's LSD test, *$P \leq 0.05$, **$P \leq 0.01$, ***$P \leq 0.001$, and ****$P \leq 0.0001$). The asterisk (*) indicates a nonspecific band.

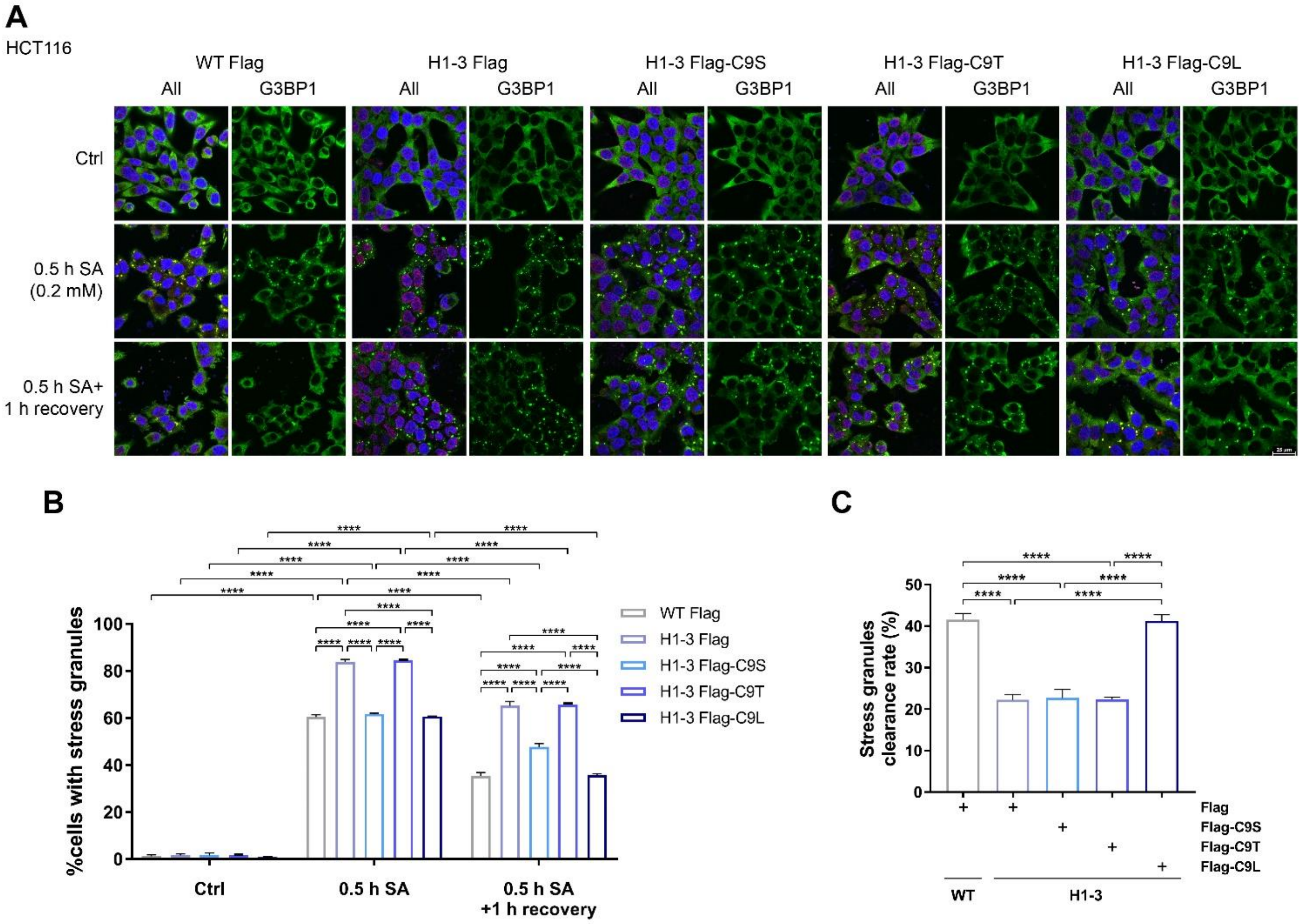

Figure S10. Reconstitution with C9S or C9L decreases SG formation and reconstitution with C9L increases the clearance of SGs in *C9orf72*$^{-/-}$ HCT116 cells. Related to Figure 6.

(A) Images of immunofluorescence staining of WT and *C9orf72*$^{-/-}$ (H1-3) HCT116 cells expressing Flag-tagged proteins treated with SA (0.2 mM) for 0.5 h with or without one hour of recovery after the removal of SA. DAPI (blue) labels the nucleus, G3BP1 (green) labels SGs, and Flag (red) labels Flag-tagged proteins. Scale bars: 25 μm. **(B)** Quantification of the percentage of cells containing SGs in HCT116 cells expressing with Flag-tagged proteins in (A) (n = 3 independent experiments, means ± SD, two-way ANOVA and followed by Tukey's

post hoc test, ****$P \leq 0.0001$); 124–212 cells were counted per condition. **(C)** Quantification of the SG clearance rate calculated from (B) (n = 3 independent experiments, means ± SD, two-way ANOVA and followed by Tukey's post hoc test, ****$P \leq 0.0001$).

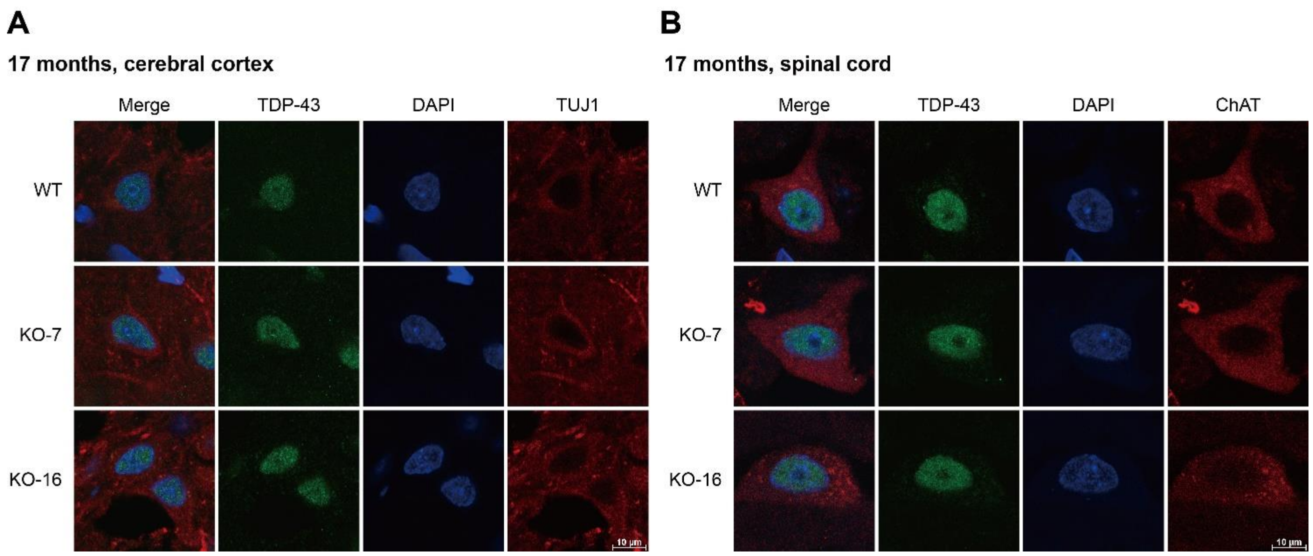

Figure S11. Loss of C9orf72 does not cause TDP-43 mislocalization in 17-month-old rat cerebral cortical neurons and spinal motor neurons.

(A) Immunofluorescence staining of the 17-month-old rat cerebral cortex labeled with endogenous TDP-43 (green) and the neuronal marker TUJ1 (red). Nuclei were counterstained with DAPI (blue). Scale bars: 10 μm. **(B)** Immunofluorescence staining of the 17-month-old rat spinal cord labeled with endogenous TDP-43 (green) and the motor neuron marker ChAT (red). Nuclei were counterstained with DAPI (blue). Scale bars: 10 μm.

Table S1. Primers used to construct GST-tagged and MBP-tagged protein expression vectors, pCS2-G3BP1-mCherry and pcDNA3.1-Flag-tagged vectors, related to the experimental procedures.

Primer name	Primer sequence (5′-3′)
GST-C9S forward	CGGGATCCATGTCGACTCTTTGCCCACC
GST-C9S reverse	CCGCTCGAGTTACTTGAGAAGAAAGCCTTC
GST-C9L forward	CGGGATCCATGTCGACTCTTTGCCCACC
GST-C9L reverse	CCGCTCGAGTTAAAAAGTCATTAGAACATC
GST-C9T forward	CGGGATCCTGTCATGAAGGCTTTCTTCTC
GST-C9T reverse	CCGCTCGAGTTAAAAAGTCATTAGAACATCTCG
MBP-eIF2S1 forward	CCGGAATTCATGCCGGGTCTAAGTTGTAG
MBP-eIF2S1 reverse	CGCGGTACCTTAATCTTCAGCTTTGGCTTCC
G3BP1-mCherry forward	AAGCTTGCCACCATGGTGATGGAGAAGCCTAGT
G3BP1-mCherry reverse	TCTAGACTATTACTTGTACAGCTCGTCCATGCC
Flag-C9S forward	ATCGGATCCATGTCGACTCTTTGCCCACC
Flag-C9S reverse	CGCGAATTCTTACTTGAGAAGAAAGCCTT
Flag-C9L forward	ATCGGATCCATGTCGACTCTTTGCCCACC
Flag-C9L reverse	CGCGAATTCTTAAAAAGTCATTAGAACATCTC
Flag-C9T forward	CGGGATCCTGTCATGAAGGCTTTCTTCTC
Flag-C9T reverse	CGCGAATTCTTAAAAAGTCATTAGAACATCTC

Table S2. Primer sequences used for semiquantitative and quantitative RT-PCR.

Gene	Forward primer sequence (5′-3′)	Reverse primer sequence (5′-3′)	Use
hEIF2S1	TGATTGAAGAAGTCGGCTCTGG	GACATAAGCCCCCATTTCAGC	RT-qPCR
hATF4	CTTCACCTTCTTACAACCTCTTCCC	GTGTAGTCTGGCTTCCTATCTCC	RT-qPCR
hCHOP	CAGAACCAGCAGAGGTCACA	CTAGCTGTGCCACTTTCCTTTC	RT-qPCR
hGRP78	GACGGGCAAAGATGTCAGGA	GCCCGTTTGGCCTTTTCTAC	RT-qPCR
hGAPDH	AGGTCGGAGTCAACGGATTTG	TGACAAGCTTCCCGTTCTCAG	RT-qPCR
rEif2s1	GCTTGCTATGGTTACGAAGGC	CATCACATACCTGGGTGGAG	RT-qPCR
rAtf4	TCTGTATGAGCCCTGAGTCCTACCT	GGTCATAAGGTTTGGGTCGAGAACCAC	RT-qPCR
rChop	CCTGAAAGCAGAAACCGGTC	CCTCATACCAGGCTTCCAGC	RT-qPCR
rXbp1s	GAGTCCGCAGCAGGTGC	GGTCCAACTTGTCCAGAATGC	RT-qPCR
rAtf6	CGAGGGAGAGGTGTCTGTTTC	GTCTTCACCTGGTCCATGAGG	RT-qPCR
rGrp78	CCTATTCCTGCGTCGGTGTATT	GGTTGGACGTGAGTTGGTTC	RT-qPCR

rAsk1	TGACACCACACAACAAGGTCT	CGAGAGGTAAGCAGATCGGC	RT-qPCR
rJnk1	ACAGAGCACCAGAGGTCATTC	GGCAAACCATTTCTCCCATAATGC	RT-qPCR
rJnk2	TGCCGATGAAACCTCGCAG	ACGCAGGCAATCCTACTGG	RT-qPCR
rTrem2	TCCTGTTGCTGGTCACAGAG	CTCCCATTCTGCTTCCTCAG	RT-qPCR
rIl-1a	GAGATTCCGGAAACACCAAA	GAAAGCTGCGGATGTGAAGT	RT-qPCR
rCcl3	CATGGCGCTCTGGAACGAA	TGCCGTCCATAGGAGAAGCA	RT-qPCR
rCcl9	GGCCCACCAGGAGGATGAA	TCTGTCGCATGTACGATCTGG	RT-qPCR
rIl-1b	CCTATGTCTTGCCCGTGGAG	CACACACTAGCAGGTCGTCA	RT-qPCR
rCxcr1	CGTTCTGGAACAGTCTGCTATG	CGGCAAGAGGAAGCCAAATA	RT-PCR
rGapdh	GGAAAGCTGTGGCGTGAT	AAGGTGGAAGAATGGGAGTT	RT-PCR & RT-qPCR
